# Revealing an Extended Adsorption/Insertion‐Filling Sodium Storage Mechanism in Petroleum Coke‐Derived Amorphous Carbon

**DOI:** 10.1002/advs.202407538

**Published:** 2024-09-16

**Authors:** Jia‐He Lv, Jing‐Song Wang, Bin He, Tao Wu, An‐Hui Lu, Wenrui Zhang, Juping Xu, Wen Yin, Guang‐Ping Hao, Wen‐Cui Li

**Affiliations:** ^1^ State Key Laboratory of Fine Chemicals School of Chemical Engineering Dalian University of Technology Dalian 116024 P. R. China; ^2^ Spallation Neutron Source Science Center Dongguan 523803 P. R. China; ^3^ Institute of High Energy Physics Chinese Academy of Sciences Beijing 100049 P. R. China

**Keywords:** closed pore filling, interlayer insertion, petroleum coke, sodium storage mechanism, sodium‐ion batteries

## Abstract

Amorphous carbon holds great promise as anode material for sodium‐ion batteries due to its cost‐effectiveness and good performance. However, its sodium storage mechanism, particularly the insertion process and origin of plateau capacity, remains controversial. Here, an extended adsorption/insertion‐filling sodium storage mechanism is proposed using petroleum coke‐derived amorphous carbon as a multi‐microcrystalline model. Combining in situ X‐ray diffraction, in situ Raman, theoretical calculations, and neutron scattering, the effective storage form and location of sodium ions in amorphous carbon are revealed. The sodium adsorption at defect sites leads to a high‐potential sloping capacity. The sodium insertion process occurs in both the pseudo‐graphite phase (*d*_002_ > 0.370 nm) and graphite‐like phase (0.345 ≤ *d*_002_ < 0.370 nm) rather than the graphite phase, contributing to low‐potential sloping capacity. The sodium filling into accessible closed pores forms quasi‐metallic sodium clusters, contributing to plateau capacity. The threshold of the effective interlayer spacing for sodium insertion is extended to 0.345 nm, breaking the consensus of insertion interlayer threshold and enhancing understanding of closed pore filling. The extended adsorption/insertion‐filling mechanism explains the sodium storage behavior of amorphous carbon with different microstructures, providing theoretical guidance for the rational design of high‐performance amorphous carbon anodes.

## Graphical abstract

The threshold of the effective interlayer spacing for sodium insertion is extended from 0.370 to 0.345 nm in petroleum coke‐derived amorphous carbon. In conjunction with the optimized structural model of amorphous carbon, an extended adsorption/insertion‐filling sodium storage mechanism is proposed, which can accurately explain the sodium storage behavior of amorphous carbon with different microstructures.

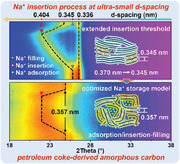

## 1 Introduction

Sodium‐ion batteries (SIBs) are a promising candidate for large‐scale energy storage due to their abundant resources, low costs, and similar to lithium‐ion batteries (LIBs) in terms of working principle and structural composition.^[^ ^1^, ^2^, ^3^, ^4^ ^]^ However, the much larger radius of Na^+^ (1.02 Å) compared to Li^+^ (0.76 Å) results in slower diffusion kinetics, presenting challenges in developing insertion‐based host material for SIBs anodes.^[^ ^5^, ^6^ ^]^ For example, graphite, highly successful as an anode material in LIBs, is unsuitable for SIBs because of the thermodynamic limitation for sodium insertion into the graphite interlayer.^[^ ^7^, ^8^, ^9^ ^]^ Fortunately, amorphous carbon, including soft carbon and hard carbon, can reversibly accommodate sodium ions and exhibit a high reversible capacity of 150–480 mAh g^−1^ owing to their large interlayer spacing and abundant active defects.^[^ ^10^, ^11^, ^12^, ^13^ ^]^

In recent years, amorphous carbons have been prepared through high‐temperature pyrolysis of various precursors, such as polymers,^[^ ^14^, ^15^ ^]^ certain biomass,^[^ ^16^, ^17^ ^]^ and coal,^[^ ^9^, ^18^ ^]^ which demonstrate excellent sodium storage properties. Amorphous carbons present different sodium storage behaviors due to their highly complex microstructure, which consists of pseudo‐graphite nanodomains, amorphous nanodomains, abundant defects, and pores with various sizes.^[^ ^19^ ^]^ In this case, the sodium storage mechanism of amorphous carbons remains elusive, especially the origin of plateau capacity and the sodium insertion process in different microstructures, which brings troubles to the structural design of carbon anodes.^[^ ^20^ ^]^

In 2000, Stevens and Dahn^[^ ^21^ ^]^ first proposed the “insertion‐filling” mechanism for sodium storage in glucose‐derived hard carbon. This mechanism was described as sodium insert into the carbon layer at the sloping region and filling into the nanopores at the plateau region.^[^ ^22^, ^23^ ^]^ In 2012, Cao et al.^[^ ^24^ ^]^ proposed an alternative “adsorption‐insertion” mechanism, positing that the sloping region corresponds to the sodium adsorption behavior on the hard carbon surface, while the plateau region arises from sodium insertion into the carbon interlayer. This study proposed that sodium easily inserts into the carbon layer with interlayer spacing larger than 0.37 nm, which has garnered support from various research groups and has progressively become the accepted threshold for sodium insertion in hard carbon.^[^ ^15^, ^25^, ^26^, ^27^ ^]^ However, Hu et al.^[^ ^28^ ^]^ didn't detect interlayer expansion during the discharge process and proposed the “adsorption‐filling” mechanism. The sloping region originates from the sodium storage on the surface, edges, and defect sites, while the plateau region corresponds to the sodium storage in nanovoids. Subsequently, Ji et al.^[^ ^29^ ^]^ comprehensively investigated the sodium storage mechanism of perylenetetracarboxylic dianhydride‐based soft carbon, suggesting that the sloping capacity primarily arises from defect adsorption and interlayer insertion. Furthermore, in situ transmission electron microscopy observed that sodium inserts into the carbon layer, causing the interlayer spacing to expand to 0.381 nm during the first sodiation. Notably, the mechanisms proposed by different researchers can only explain the sodium storage behavior of specific amorphous carbons, and no consensus has been reached on the sodium storage mechanism of amorphous carbons.

Furthermore, in recent investigations of soft carbon, it has been observed that sodium can readily insert into the carbon layer with an interlayer spacing of < 0.37 nm,^[^ ^19^, ^29^, ^30^ ^]^ even when reduced to 0.35 nm.^[^ ^19^ ^]^ However, theoretical energy calculations based on existing sodium storage models of hard carbon suggest the existence of insurmountable energy barriers for this process.^[^ ^24^, ^25^, ^26^ ^]^ In other words, the discussions on the impact of carbon interlayer spacing on sodium insertion into hard carbon may not be applicable to soft carbon. This discovery opens the door for the preparation of commercial anode materials for SIBs with excellent performance using low‐cost and high‐yield soft carbon precursors such as pitch and petroleum coke.^[^ ^31^, ^32^, ^33^, ^34^ ^]^ However, not all layers can facilitate sodium insertion, such as graphite. Therefore, it is imperative to elucidate the impact of interlayer spacing on insertion to provide guidance for the structural optimization of soft carbon. In past decades, the consensus reached on the interlayer spacing of sodium insertion is that sodium cannot insert into carbon layers with *d*_002_ = 0.335 nm (as in graphite), while sodium can freely insert into carbon layers with *d*_002_ > 0.370 nm (as in hard carbon). However, studies regarding sodium insertion into the carbon interlayer with interlayer spacing in the range of 0.335–0.370 nm are scarce and highly anticipated.

To comprehensively understand the sodium storage mechanism of amorphous carbon, a series of amorphous carbon samples with different interlayer spacing and multi‐microcrystalline coexistence were prepared by varying the pyrolysis temperature, using petroleum coke (PC) as the raw material. Petroleum coke is recognized as a high‐quality precursor for energy storage applications because of its high electrical conductivity, ease of graphitization, and high carbon content,^[^ ^35^ ^]^ such as graphite for LIBs and superactivated carbon for supercapacitors. Furthermore, The multi‐microcrystalline coexistence within the petroleum coke allows us to independently study the effect of carbon interlayer spacing on sodium insertion and filling behavior in a controlled microenvironment and provide guidance for optimizing PC‐based anode material with low sodium storage capacity. In this work, combining experiments and theoretical calculations, the effect of the microstructure of PC‐based carbon on sodium storage behavior was revealed. The results indicate that the sodium insertion behavior in the pseudo‐graphite phase (*d*_002_ > 0.370 nm) and the graphite‐like phase (0.345 ≤ *d*_002_ < 0.370 nm) rather than the graphite phase, along with adsorption behavior, contribute to the sloping capacity. The plateau capacity primarily results from the filling of accessible closed pores, which form quasi‐metallic sodium clusters. The presence of the graphite phase does not effectively facilitate the insertion and transportation of sodium ions and reduces the accessible closed pore volume, so it should be prevented during material preparation. The extended adsorption/insertion‐filling mechanism extends the effective interlayer spacing for sodium insertion to 0.345 nm, effectively addressing the origin of plateau capacity and interlayer threshold. This provides valuable guidance for the development of cost‐effective and high‐performance carbon anode materials for SIBs.

## 2 Results and Discussion

**Figure 1:**
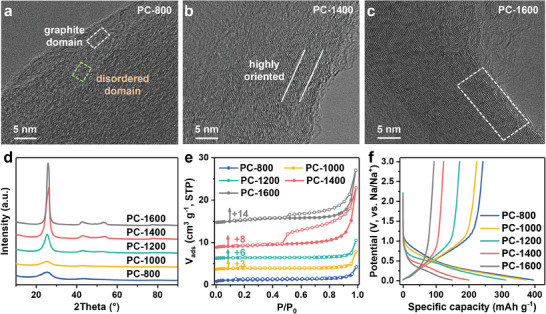
HRTEM images of a) PC‐800, b) PC‐1400, c) PC‐1600. d) XRD patterns of PC‐T. e) N_2_ adsorption‐desorption isotherms of PC‐T, +x represents the number of units by which the isotherm is shifted upward. f) the initial galvanostatic charge/discharge curves of PC‐T.

A series of amorphous carbons were prepared by direct pyrolysis of PC in the temperature range of 800–1600 °C for 2 h and denoted as PC‐T (*T* represents the pyrolysis temperature). As shown in high‐resolution transmission electron microscopy (HRTEM, **Figure** **1**a–c), PC‐800 shows an amorphous structure consisting of graphite domains and disordered domains. With increasing pyrolysis temperature, the alignment and orientation of the graphite domain in PC‐T samples are enhanced. In this case, the PC‐1400 and PC‐1600 exhibit typical semi graphitic structures of soft carbon.^[^ ^19^ ^]^ Additionally, the multi‐microcrystalline structure of PC‐T (Figure S1, Supporting Information) confirms the feasibility of PC‐derived carbon as a model for investigating the effect of interlayer spacing on sodium insertion. Two broad diffraction signals at 23° and 43° appear in the X‐ray diffraction (XRD) patterns (Figure 1d) of PC‐T samples, corresponding to the (002) and (100) diffractions of the graphite‐like domains in carbon materials.^[^ ^36^, ^37^ ^]^ With increasing pyrolysis temperature, the (002) reflection shows obvious narrowing and right‐shift, indicating higher graphitization and smaller interlayer spacing. Based on the Bragg equation and Debye‐Scherrer equation,^[^ ^38^ ^]^ the interlayer spacing (*d*_002_), average width (*L*_a_), and average thickness (*L*_c_) of carbon microcrystallites were calculated as shown in Figure S2a and Table S2 (Supporting Information). The *d*_002_ of all samples are in the range of 0.335–0.370 nm. Moreover, the values of *L*_c_ and *L*_a_ exhibit an increasing trend with the increasing pyrolysis temperature, indicating the regulation of carbon microstructure by pyrolysis at different temperatures.

The specific surface area and pore structure of PC‐T samples were evaluated using N_2_ adsorption‐desorption measurements (Figure 1e; Figure S2b, Supporting Information). All PC‐T samples exhibit an ultra‐low specific surface area based on the Barrett‐Emmett‐Teller method (*S*_BET_ < 5 m^2^ g^−1^), with pore size distribution primarily in the mesoporous range. Note that the gas adsorption‐desorption method is only sensitive to open pores accessible to gas, but is not applicable for the analysis of closed pores. To further analyze the closed pores in the PC‐T samples, the true density analysis based on Archimedes' principle was conducted using helium (He) as a molecular probe.^[^ ^6^ ^]^ As shown in Figure S2c (Supporting Information), closed pores are detected in all PC‐T samples, and the closed pore volume (CPV) exhibits a trend of decreasing and then increasing with the increase of pyrolysis temperature. The decrease in CPV is primarily attributed to the rearrangement and directional alignment of the carbon layer, which occupies the primary pores.^[^ ^19^ ^]^ As the temperature increases further, the increase in CPV is mainly due to the decomposition of sulfur‐containing compounds, which release gas and form additional pores. The structural parameters of the PC‐derived carbon are summarized in Table S2 (Supporting Information), suggesting that a series of amorphous carbons with different microcrystalline parameters were successfully synthesized.

The sodium storage behavior of the PC‐T samples was evaluated using Na half‐cells in the voltage range of 0.01‐3 V. The galvanostatic charge/discharge (GCD) curves of PC‐T at 20 mA g^−1^ are shown in Figure 1f. All PC‐T samples show typical sodium storage behavior of soft carbon with a prominent sloping region above 0.1 V and negligible plateau region below 0.1 V. The sloping capacity of PC‐T for the first charge gradually decreases (Figure S3, Supporting Information), which is caused by the fewer available defect sites for sodium adsorption with increasing pyrolysis temperature. However, the plateau capacity for all PC‐T samples maintains low (< 23 mAh g^−1^), even for all PC‐T samples exhibiting closed pores. These results raise the question of whether the plateau capacity is associated with the sodium filling in closed pores. Additionally, it is a wonder that all PC‐T samples exhibit low initial Coulombic efficiency (ICE) despite their low *S*_BET_, a phenomenon that will be explained later.

**Figure 2:**
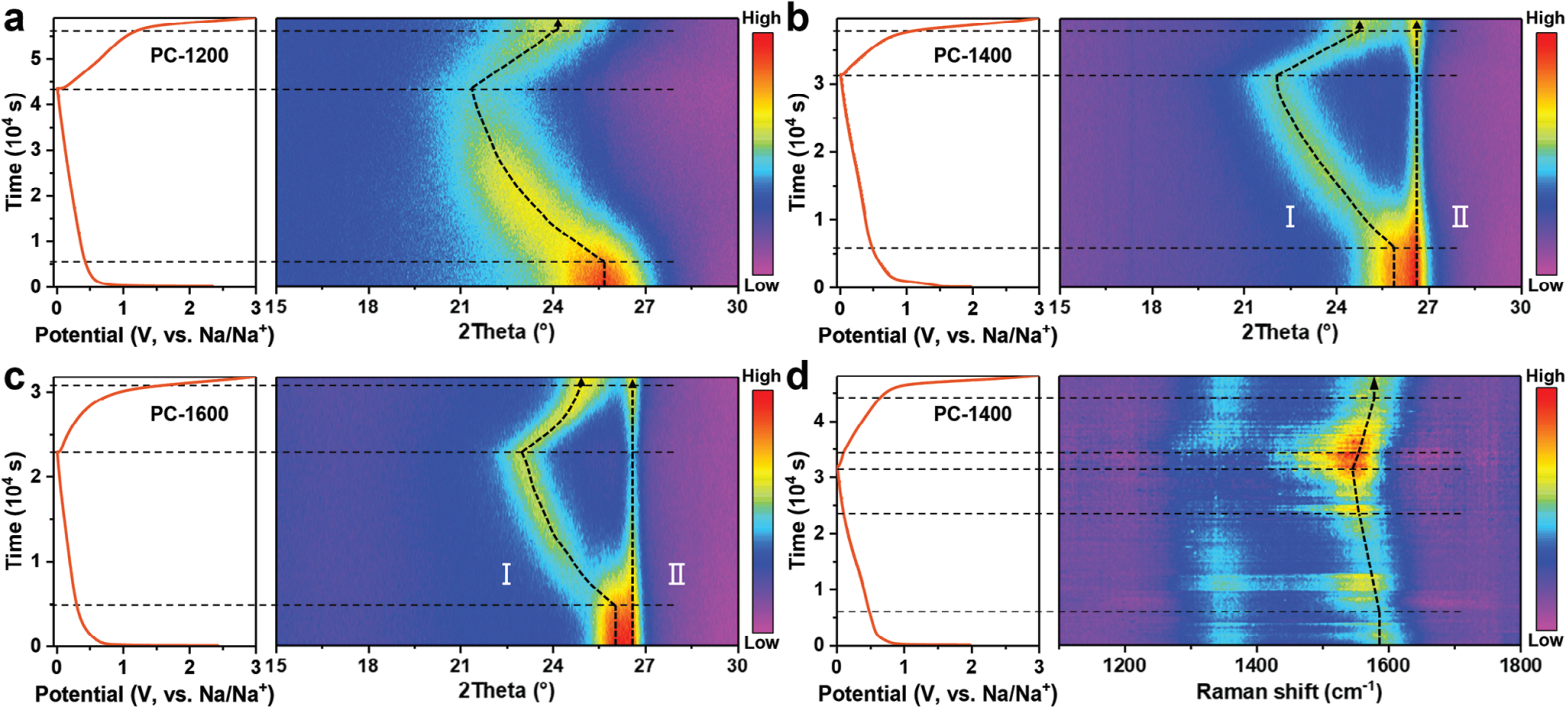
The in situ XRD patterns of the first cycle at 25 mA g^−1^ for a) PC‐1200, b) PC‐1400, and c) PC‐1600 and d) the in situ Raman mapping of the first cycle at 25 mA g^−1^ for PC‐1400.

To understand the above‐mentioned issues deeply and systematically, time‐resolved in situ XRD was employed to effectively monitor the changes in interlayer spacing during sodium storage. It should be mentioned that the narrow and strong (002) reflection (Figure 1d) appears in the PC‐T samples (*T* > 1000 °C), which can ensure more precise and sensitive detection of structural changes for XRD test during sodium storage. The recorded in situ XRD patterns of PC‐1200, PC‐1400, and PC‐1600 with the corresponding initial discharge/charge curves are displayed in **Figure** **2**. There is no obvious shift in the (002) reflection of the three samples during the initial discharge process above 0.5 V, indicating sodium adsorption behavior rather than insertion behavior in this region. With continuous discharge to below 0.5 V, the obvious left shifts of the (002) reflection confirm sodium insertion into the carbon interlayers.^[^ ^19^ ^]^ With subsequent charging, the (002) reflection of PC‐1200, PC‐1400, and PC‐1600 doesn't recover to the original position, indicating that some sodium ions are permanently trapped in the graphitic‐like microcrystallites.^[^ ^22^ ^]^ The irreversible sodium insertion into the interlayers is the main reason for low ICE for PC‐T samples.

Additionally, it is noticed that the (002) reflection of the PC‐1200 sample shifts from 25.6 ° (0.348 nm) to 21.3 ° (0.417 nm) during the discharge process, demonstrating that the sodium insertion process can still occur in carbon layers below 0.370 nm. For the PC‐1400 and PC‐1600 with high crystallinity, a left‐shift and splitting of the (002) reflection can be observed simultaneously, which indicates that the sodium insertion process in the multi‐microcrystalline PC‐derived carbon is selective. Reflection I (25.8 °) shifts to a lower angle, evidencing the expansion of carbon layers with interlayer spacing of 0.345 nm, while the reflection II position (26.5°) remains unchanged, indicating that there is no sodium insertion in the graphite domain (0.336 nm). This result is further confirmed by in situ XRD analysis of graphite with interlayer spacing of 0.336 nm (Figure S4, Supporting Information). Furthermore, a similar left‐shift (reflection II, 0.345 nm) and splitting of the (002) reflection is observed in the in situ XRD of PA‐1600 (Petroleum asphalt pyrolyzed at 1600 °C, Figure S5, Supporting Information), indicating that the sodium insertion process at ultra‐small interlayer spacing is universal. These results prove that the sodium insertion process is highly dependent on the carbon interlayer spacing and can occur in the carbon domain with interlayer spacing ≥ 0.345 nm.

In situ Raman spectroscopy was conducted to further investigate the sodium storage behavior of PC‐derived carbon (Figure 2d), providing additional information complementary to the XRD results. Two characteristic peaks appear in the Raman spectrum, including the defect‐induced D‐band at ≈1350 cm^−1^ and the in‐plane vibration G‐band of crystalline graphite at ≈1580 cm^−1^. During the initial sodiation process (above 0.5 V), the G‐band position remains unchanged, while the D‐band intensity gradually weakens due to the sodium adsorption at defects and pores, which restricts breathing vibration of sp^2^ carbon ring at defects and the edge of the carbon layer.^[^ ^37^ ^]^ Therefore, sodium adsorption is the primarily sodium storage mechanism at high potential. As sodiation continues below 0.5 V, the G‐band gradually shifts to the left. The phenomenon is attributed to sodium insertion into the carbon layer, which leads to electron occupation in the π* antibonding‐band, lengthening and weakening the C─C bond, and thus causing a redshift of the G‐band.^[^ ^15^ ^]^ Notably, the D‐band intensity significantly weakens between 0.1 and 0.01 V, corresponding to sodium adsorption on the closed pore surface or pore filling by Na clusters.^[^ ^6^ ^]^ The Raman results align with the XRD findings, which not only confirm the sodium insertion process in ultra‐small interlayer spacing but also provide visual evidence for the closed pores filling process.

**Figure 3:**
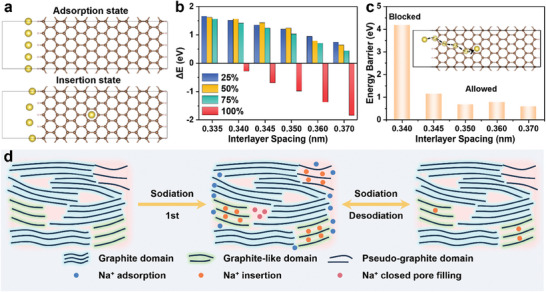
The calculation models of a) sodium adsorption and insertion state as the edge coverage reaches 100%. b) Δ*E* values of adsorption‐insertion state transition with interlayer spacing at different edge coverages. c) The energy barriers for the adsorption‐insertion state transition with different interlayer spacing (inset: The sodium ions diffusion path in the carbon layer). d) Schematic illustration of the microstructure‐dependent sodium storage mechanism of PC‐derived carbon.

To further evaluate the impact of carbon interlayer spacing on sodium insertion behavior, density functional theory (DFT) calculations were performed on a series of models with varying interlayer spacings. The edge coverage of sodium ions was introduced for further discussion, as detailed in the supporting information (Figure S6, Supporting Information). The adsorption and insertion states were simulated with edge coverages of 25, 50, 75, and 100%, and the optimized results were shown in **Figures** **3**a and S7 (Supporting Information). Since the sodium adsorption precedes interlayer insertion during sodium storage, the Δ*E* is calculated to evaluate the thermodynamic feasibility of the adsorption‐insertion state transition using the formula below:

(1) $$\Delta E=E_{\mathrm{ins}}-E_{\mathrm{ads}}$$

Where *E*_ins_ and *E*_ads_ are the calculated total energy of the insertion state and adsorption state, respectively. The calculated results are presented in Figure 3b, where Δ*E* for all layer spacings are >0 eV when edge coverages are < 100%, indicating that the adsorption‐insertion state transition cannot occur spontaneously. As the edge coverage reaches 100%, Δ*E* gradually decreases from −0.02 eV (0.335 nm) to −1.85 eV (0.370 nm) with increasing interlayer spacing. Compared to graphite (0.335 nm) with Δ*E* close to 0 eV, models with interlayer spacing ≥ 0.340 nm exhibit Δ*E* far from 0 eV, indicating a higher likelihood of sodium insertion. These results also suggest a strict sequential order in sodium storage behavior, progressing from sodium adsorption to insertion, consistent with previous experimental findings. Additionally, the diffusion process of the adsorption‐insertion state transition for various interlayer spacings >0.340 nm was simulated using the climbing image nudged elastic band (CI‐NEB) method. The sodium ions diffusion paths at different layer spacings are illustrated in Figures S8–S12 (Supporting Information). The intermediate state energies exhibit fluctuations due to differences in sodium ions diffusion paths as depicted in Figure S13 (Supporting Information). The energy barriers of sodium insertion into carbon models with different interlayer spacing are calculated by comparing the intermediate state energy of two steps (Figure 3c). At an interlayer spacing of 0.340 nm, sodium ions diffusion is highly unstable with an energy barrier as high as 4.18 eV, indicating the diffusion process is difficult. However, with increasing interlayer spacing, the energy barrier rapidly decreases to 1.14 eV at 0.345 nm and stabilizes ≈0.6 eV in the range of 0.350–0.370 nm, facilitating an easier diffusion process. Theoretical calculations demonstrate that the adsorption‐insertion state transition in the carbon layer with interlayer spacing ≥ 0.345 nm is both thermodynamically and dynamically feasible, consistent with observations from in situ XRD results. The threshold of the effective interlayer spacing for sodium insertion is extended to 0.345 nm, which breaks through the consensus of the threshold of insertion interlayer (0.370 nm) and provides a reference for the analysis of effective sodium storage in closed pores. Based on the *d*_002_ values, the carbon phase can be divided into three types: pseudo‐graphite phase (*d*_002_ > 0.370 nm), graphite‐like phase (0.345 ≤ *d*_002_ < 0.370 nm), and graphite phase.^[^ ^39^ ^]^

Based on the above experimental results and analysis, we speculate that the sodium filling into closed pores is related to the carbon microcrystalline states around the closed pores.^[^ ^40^ ^]^ sodium can only diffuse into the closed pores through the surrounding graphite‐like and pseudo‐graphite phases, which are defined as accessible closed pores. To identify the microcrystalline phase, the (002) reflection of PC‐T is simulated by a profile‐fitting process (Figure S14 and Table S3, Supporting Information).^[^ ^41^ ^]^ The accessible closed pore volume (ACPV) calculated by multiplying the total CPV by the ratio of pseudo‐graphite and graphite‐like phases in the microcrystalline phase are all low (Figure S15a, Supporting Information). As shown in Figure S15b (Supporting Information), the plateau capacity and ACPV of PC‐T follow the linear equation *Y* = 1128.6 *X*, with a correlation coefficient of 0.99. The slope is very close to the theoretical specific capacity of metallic sodium (1128 mAh cm^−3^), indicating that the plateau capacity is closely related to ACPV. Based on the above analysis, a microstructure‐dependent sodium storage mechanism of PC‐T is proposed in Figure 3d, including three distinct sodium storage behaviors: sodium adsorption at defective sites, sodium insertion within the pseudo‐graphite and graphite‐like phases, and sodium filling into the accessible closed pore. Additionally, some sodium ions can be trapped by the carbon layer during the desodiation process, leading to low ICE. The presence of graphite domains does not effectively facilitate sodium insertion and therefore should be prevented in the material preparation process.

**Figure 4:**
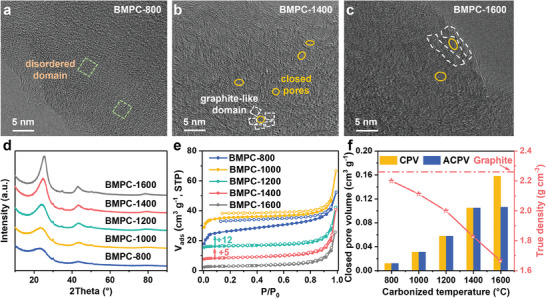
HRTEM images of a) BMPC‐800, b) BMPC‐1400, c) BMPC‐1600. d) XRD patterns of BMPC‐T. e) N_2_ adsorption‐desorption isotherms of BMPC‐T, +x represents the number of units by which the isotherm is shifted upward. f) CPV, ACPV, and true density of BMPC‐T.

To verify the rationality of the microstructure‐dependent sodium storage mechanism, the microcrystalline structure was optimized using high‐energy ball milling followed by pyrolysis to reduce the graphite phase content and increase the ACPV, which is expected to improve the electrochemical performance of BMPC‐derived carbon. Figures S16 and S17 (Supporting Information) demonstrate that ball milling treatment reduces the particle size and graphitization of PC, and introduces oxygen‐containing functional groups on its surface. The effect of ball milling on the structure of PC stabilizes after 3 h, so PC‐3 h is selected as the precursor for subsequent discussion. The N_2_ adsorption‐desorption isotherms of petroleum coke after ball milling treatment(Figure S17c, Supporting Information) show that ball milling treatment can open previously closed pores in the petroleum coke, increasing its specific surface area from 1.6 to 46.1 m^2^ g^−1^. As shown in Figure S17d (Supporting Information), the thermogravimetric analysis (TG) results show that PC‐3 h exhibits a lower carbon yield and weight loss temperature compared to PC, suggesting that ball milling reduces the thermal stability of PC. A series of BMPC‐T samples were synthesized by pyrolyzing PC‐3 h within the temperature range of 800—1600 °C. The HRTEM results (**Figure** **4**a) show that the BMPC‐800 exhibits a highly disordered microcrystalline structure. With increasing pyrolysis temperature, the random stacking and limited rearrangement of the carbon layers lead to the formation of a turbostratic structure with closed pores and locally twisted domains, characteristic of the hard carbon structure. The range of graphite‐like domains and the size of the closed pores are also increasing. The XRD results (Figure 4d) demonstrate that the (002) reflection of BMPC‐T is wider and weaker than PC‐T, indicating a more disordered structure in BMPC‐T. The (002) reflection fitting results for the BMPC‐T (Figure S19, Supporting Information) reveal that graphite‐like and pseudo‐graphite phases are the main components of the sample, and the graphite phase appears only in the BMPC‐1600 sample. The ball milling followed by pyrolysis treatment successfully converts soft carbon into hard carbon, significantly reducing the content of graphite phases.

The porosity parameters of the BMPC‐T samples were characterized by N_2_ adsorption‐desorption measurements as shown in Figure 4e. The high specific surface area of BMPC‐800 (103 m^2^ g^−1^) and BMPC‐1000 (142 m^2^ g^−1^) is primarily attributed to the release of small molecules to form some defects and open pores. With increasing pyrolysis temperature, the growth of carbon domains and the decrease of defects cause a rapid decrease in the *S*_BET_ of BMPC‐T (*T* > 1000 °C; *S*_BET_ < 13 m^2^ g^−1^). In addition, the true density and closed pore volume of the BMPC‐T samples are analyzed in Figure 4f. With increasing pyrolysis temperature, the true density values of BMPC‐T gradually decrease, while the corresponding closed pore volume gradually increases. This is attributed to the transformation of some open pores into closed pores due to the rearrangement of the carbon layers and the growth of the microcrystalline structure. Since only the BMPC‐1600 sample contains graphite phases, its ACPV is lower than its CPV. SAXS results (Figure S18c, Supporting Information) show that BMPC‐1400 exhibits a more pronounced peak at ≈0.1 Å^−1^ compared to BMPC‐800, which is a typical characteristic of closed pores.^[^ ^42^ ^]^ The structural parameters of the BMPC‐T samples are summarized in Table S2 (Supporting Information), demonstrating the successful preparation of PC‐based hard carbon with large interlayer spacing and abundant accessible closed pores.

**Figure 5:**
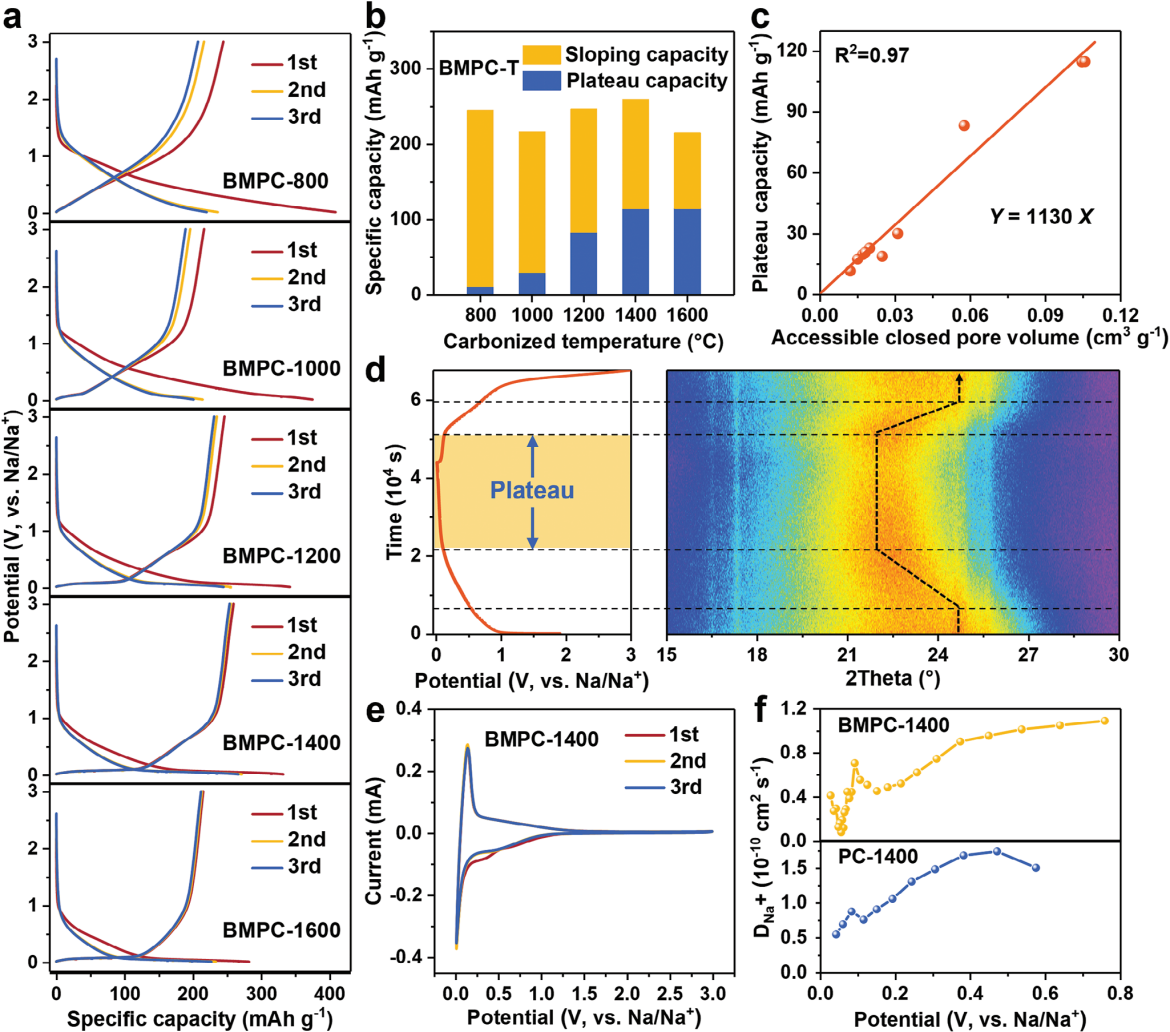
a) Charge/discharge profiles of the initial three cycles and b) the sloping capacity and plateau capacity of BMPC‐T samples at 20 mA g^−1^. c) Correlation curves between the plateau capacity and ACPV. d) The in situ XRD patterns of the first cycle at 25 mA g^−1^ for BMPC‐1400. e) CV curves of BMPC‐1400 at a scan rate of 0.1 mV s^−1^. f) The variation of calculated sodium ions diffusion coefficients against different potentials for BMPC‐1400 and PC‐1400.

The electrochemical performance of BMPC‐T samples was evaluated in a half‐cell. There is only a sloping region without an obvious plateau region in BMPC‐800 and BMPC‐1000 (**Figure** **5**a). The plateau region appears when the pyrolysis temperature increases to 1200 °C, corresponding to the sample with abundant closed pores. The sloping capacity of BMPC‐T for the first charge gradually decreases with increasing pyrolysis temperature (Figure 5b), which is consistent with the decreasing trend of surface defects and interlayer spacing in the sample. This further confirms that the sloping capacity is derived from the adsorption/insertion process of sodium ions. The plateau capacity first increases and then decreases, reaching a maximum at 1400 °C. The reduction of the interlayer spacing below 0.370 nm for the BMPC‐T samples does not affect the closed pore filling process, which further validates the rationality of the idea of extending the interlayer spacing threshold. The BMPC‐1400 exhibits the highest reversible capacity of 259.8 mAh g^−1^ with enhanced ICE (78.1%) and plateau capacity (114.6 mAh g^−1^). The strong linear correlation between the ACPV and plateau capacity for all samples is depicted in Figure 5c, further confirming that the plateau capacity originates from sodium filling into the accessible closed pore. BMPC‐T with large interlayer spacing and abundant ACPV demonstrates a high plateau capacity, confirming the rationality of the microstructure‐dependent sodium storage mechanism and the correspondence between accessible closed pores and plateau capacity.

In situ XRD was employed to investigate the structural changes of the BMPC‐1400 during initial sodiation and desodiation. The (002) reflection position of BMPC‐1400 shows a similar trend in the sloping region as that of the PC‐T sample, corresponding to the sodium adsorption and then insertion behavior. For the plateau region (below 0.1 V), the (002) reflection position of BMPC‐1400 shows no significant shift, suggesting closed pores filling. During the desodiation process, the (002) reflection gradually returns to the original angle, indicating that the expanded interlayer spacing facilitates the reversible insertion and extraction of sodium ions, consistent with its high ICE. The sodium storage behavior and kinetic properties of BMPC‐T were further characterized by cyclic voltammetry (CV) and galvanostatic intermittent titration technique (GITT). As shown in Figure 5e and Figure S21 (Supporting Information), two irreversible peaks at 0.75 and 0.25 V in the first cathodic scan attributed to electrolyte decomposition forming the solid electrolyte interphase (SEI) layer and irreversible sodium insertion between carbon layers, respectively.^[^ ^29^ ^]^ Compared to PC‐1400 and BMPC‐800, a sharper redox peak appears at ≈0.01 V in BMPC‐1400, which is consistent with the higher plateau capacity observed in BMPC‐1400. Based on the power‐law relationship between scan rate (*v*) and peak current (*i*): *i* = a*v* ^b^, the sodium storage behavior of the material can be determined by the calculated b value (Figure S22, Supporting Information),^[^ ^43^ ^]^ where b = 0.5 indicates a diffusion‐controlled behavior and b = 1 indicates a capacitive‐controlled behavior. Compared to PC‐1400 (b = 0.69) and BMPC‐800 (b = 0.67), the b value of BMPC‐1400 (b = 0.53) is closer to 0.5, suggesting that the plateau region is dominated by diffusion‐controlled behavior.

To further understand the difference in the kinetic properties between BMPC‐1400, PC‐1400, and BMPC‐800, the apparent diffusion coefficient of sodium ions (*D*_Na_^+^) was evaluated by GITT. As shown in Figure 5f and Figure S23 (Supporting Information), the *D*_Na_^+^ of all samples is higher in the sloping region, corresponding to sodium adsorption at the surface and defect sites. As the potential decreases to 0.1 V, there is a significant decrease in *D*_Na_^+^, which corresponds to sodium insertion into carbon layers. Below 0.1 V, the *D*_Na_^+^ of BMPC‐800 and PC‐1400 continues to decrease, while BMPC‐1400 exhibits a rapid decline at 0.1 V, followed by an increase near the cut‐off voltage, indicating a distinct sodium storage behavior in the plateau region for the three samples. The increase in *D*_Na_^+^ near the cut‐off voltage is often attributed to the clustering of sodium ions in closed pores.^[^ ^44^, ^45^, ^46^ ^]^ The low *D*_Na_^+^ is mainly observed in the plateau region, providing insight into the rapid fading of plateau capacity at high current density. The rate performance (Figure S24, Supporting Information) reveals that the reversible capacity of BMPC‐1400 with high plateau capacity decreases rapidly with increasing current density. The reversible capacity decay primarily results from the rapid loss of the plateau capacity. Materials with a higher accessible closed pore content experience faster capacity loss, which further suggests that the plateau capacity originates from the closed pore filling process with sluggish dynamics. Moreover, to determine the state of sodium in the closed pores, the electrodes after discharge to 0.01 V are soaked in an ethanol solution containing 1% phenolphthalein (Figure S25, Supporting Information). Only the solution soaked with BMPC‐1400 turned red, indicating the sodium in the closed pore formed quasi‐metallic sodium clusters.^[^ ^47^, ^48^ ^]^

**Figure 6:**
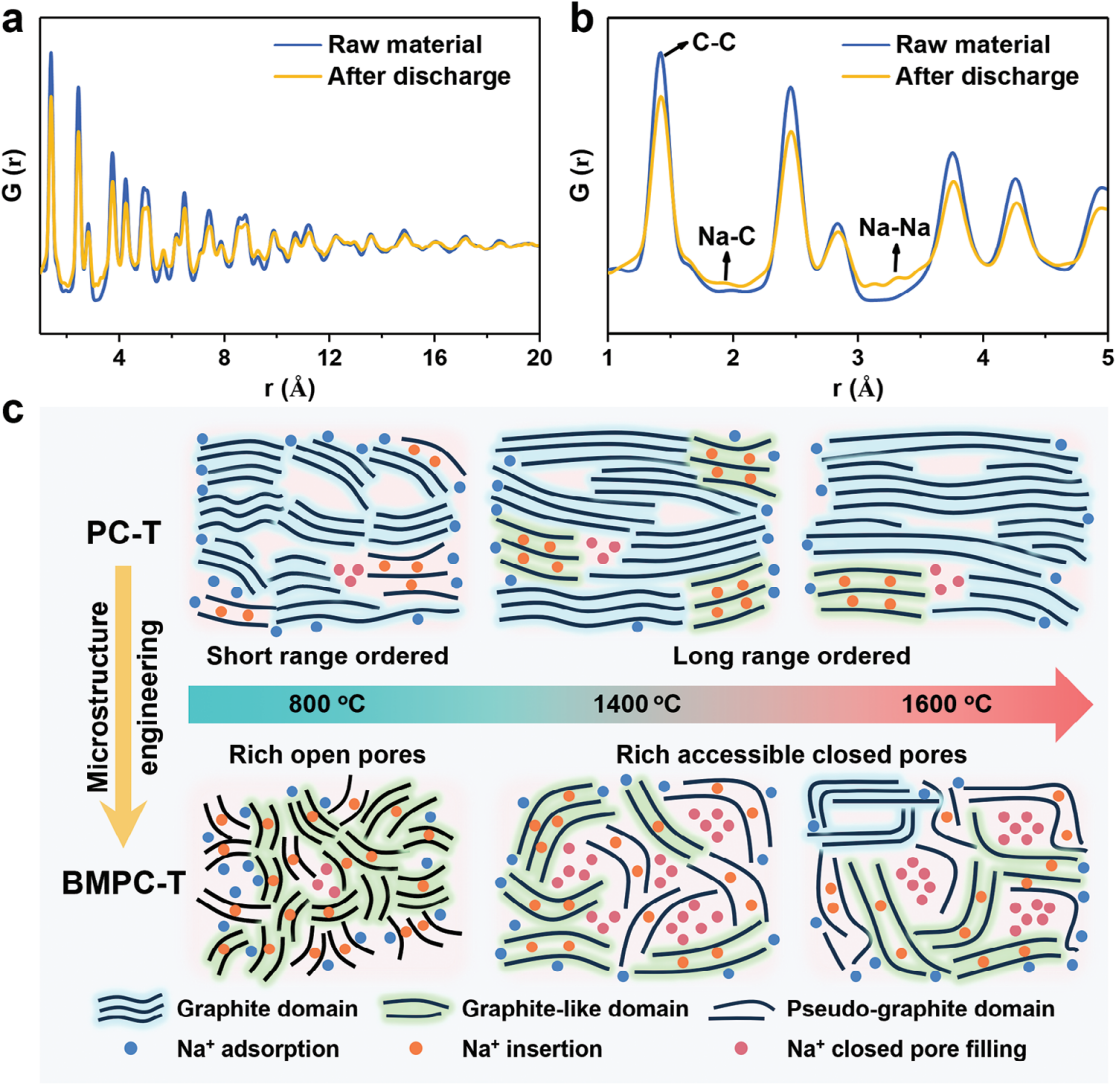
PDF results of neutron total scattering of BMPC‐1400 raw material and after discharge: a) a long‐range profile and b) the expanded short‐range profile. c) Schematic representation of the microstructure and sodium storage mechanism for PC‐derived carbon synthesized at different temperatures.

The pair distribution function (PDF) method is sensitive to light elements (e.g., Na, Mg, and Al) and their adjacent counterparts, making it a powerful tool for analyzing the interaction between Na and C. Generally when the electron transfers to the carbon layers along with the insertion of alkali metal ions, it will cause the occupation of π* antibonding‐band to make the C─C bond weaken and lengthen.^[^ ^6^ ^]^ As shown in **Figure** **6**a, the main peak of the discharged sample becomes lower, indicating that the atomic number density within this radius is significantly lower than the average density, and the mutual bonding strength between the carbon atoms is also relatively weak. These results indicate the sodium insertion into carbon layers. The faint peak around 1.9 Å also suggests the formation of Na─C bonds corresponding to the sodium insertion process.^[^ ^49^ ^]^ Additionally, the peak around 3.0–3.5 Å may correspond to Na─Na bonds, arising from quasi‐metallic sodium clusters formed in the closed pore.^[^ ^50^ ^]^ Combining neutron scattering, structural characterization, and electrochemical characterization results, the effective storage form and location of sodium ions in PC‐based carbon can be identified: 1) the adsorption of sodium ions on the surface and defective sites; 2) sodium insertion into non‐graphite carbon layers; 3) sodium filling into the accessible closed pore to form quasi‐metallic sodium clusters.

Based on the above analysis, the temperature‐dependent structure and sodium storage behavior of PC‐derived carbon is modelled in Figure 6c, and an extended adsorption/insertion‐filling mechanism is proposed. The combined contribution from surface/defect adsorption and interlayer insertion to the sloping capacity is considered to operate for both PC‐based soft carbons and hard carbons. Additionally, the sodium insertion process occurs in both graphite‐like and pseudo‐graphite phases. Graphite phases with small layer spacing are considered incapable of storing sodium. The plateau capacity originates from the sodium filling into the accessible closed pore, forming quasi‐metallic sodium clusters. With increasing pyrolysis temperature, the gradual decrease in non‐graphite phase and defects leads to a gradual decrease in sodium adsorption and sodium insertion sites, resulting in a gradual decrease in the sloping capacity of PC‐T and BMPC‐T. PC‐T exhibits a low plateau capacity due to the high content of the graphite phase and limited ACPV. BMPC‐T exhibits abundant sodium ions diffusion channels and accessible closed pores, contributing to its high plateau capacity. This part of the study offers valuable insights into the practical application of soft carbon precursors for SIBs.

## 3 Conclusion

In summary, we have prepared a series of PC‐based amorphous carbon by varying the pyrolysis temperature, which is used as model materials to investigate the sodium storage mechanism using in situ XRD, in situ Raman, multiangle electrochemical analyses, theoretical calculations, and neutron scattering. We proposed an extended adsorption/insertion‐filling mechanism: the sloping capacity of PC‐based amorphous carbon is contributed by the sodium adsorption and interlayer insertion, while the plateau capacity is primarily attributed to sodium filling into the accessible closed pore to form quasi‐metallic sodium clusters. Importantly, the sodium insertion process is highly dependent on the interlayer spacing, and it can occur in the pseudo‐graphite (*d*_002_ > 0.370 nm) and graphite‐like (0.345 ≤ *d*_002_ < 0.370 nm) phases but not in the graphite phase. Moreover, the concept of accessible closed pores is introduced to evaluate the contribution of closed pores to plateau capacity. This analysis addresses the puzzling observation that some carbon materials with abundant closed pores exhibit low plateau capacity. This work will offer a new perspective on the sodium storage mechanism of carbon anode for SIBs.

## Conflict of Interest

The authors declare no conflict of interest.

## Supporting information



Supporting Information

## Data Availability

The data that support the findings of this study are available in the supplementary material of this article.
