# Supplementary material for: Revealing an Extended Adsorption/Insertion‐Filling Sodium Storage Mechanism in Petroleum Coke‐Derived Amorphous Carbon
Source: Adv Sci (Weinh). 2024 Sep 16;11(42):2407538. doi: 10.1002/advs.202407538 (PMC11558153; doi:10.1002/advs.202407538)
Supplement: Supplementary file 1 — Supporting Information [file ADVS-11-2407538-s001.docx]

Supporting Information

**Revealing an** **Extended Adsorption/Insertion-Filling Sodium Storage Mechanism in Petroleum Coke-Derived Amorphous Carbon**

*Jia-He Lv, Jing-Song Wang, Bin He, Tao Wu, An-Hui Lu, Wenrui Zhang, Juping Xu, Wen Yin, Guang-Ping Hao, Wen-Cui Li**

**Experimental Section/Methods**

*Material Synthesis*

The pristine petroleum coke (PC, Qingdao, Shandong Province) was pyrolysis at different temperatures of 800-1600 °C for 2 h in a tubular furnace under argon atmosphere with a heating rate of 4 ℃ min^-1^. The obtained samples were denoted as PC-T (*T* refers to the pyrolysis temperature, *T* = 800, 1000, 1200, 1400, 1600 ℃). To optimize the microstructure of PC, PC was treated by high-energy ball milling at 800 rpm to obtain ball milling PC, noted as PC-x (*x* refers to the ball milling time, *x* = 0.1, 1, 2, 3, 4 h). Additionally, BMPC-T were prepared by treating the PC-3h using the same pyrolysis procedure as that for PC-T. To prove the applicability of the proposed sodium storage mechanism to different precursors, PA-1600 was prepared by pyrolysis of medium-temperature petroleum asphalt at 1600 °C for 2 h with a heating rate of 4 ℃ min^-1^.

*Materials Characterizations*

High-resolution transmission electron microscopy (HRTEM, JEM-F200) and Optical microscopy (Nikon, LV100ND) were used to characterize the morphologies and microstructure of the as-prepared samples. The crystal structure was investigated by X-ray diffraction pattern (XRD, PANalytical X’Pert 3) with a Cu *K*α radiation (*λ* = 0.15418 nm) and Raman spectroscopy (Raman, Thermo Fisher Scientific DXR Raman microscope) with a 532 nm laser excitation. FTIR (Nicolet 6700) was used for speculation on chemical bonding information. The pyrolysis behavior of petroleum coke was analyzed by thermogravimetric (TG) equipment. TG pyrolysis experiments were performed at a heating rate of 10 °C/min in a temperature range of 25-1300 °C. The nitrogen adsorption-desorption isotherms were obtained using a Micromeritics Tristar 3000 device at 77 K. The specific surface areas (*S*_BET_) were estimated by the Barrett-Emmett-Teller (BET) method. The true density was analyzed using an AccuPyc 1345 instrument with helium as the analysis gas. Helium was chosen for this purpose due to its ultra-small aerodynamic diameter, enabling it to penetrate almost all the open pores of the materials. The small angle X-ray scattering (SAXS, Xeuss 2.0) was carried out to study the closed pores of as-prepared samples. Neutron pair distribution function (nPDF) experiments were carried out on Multi-Physics Instrument (MPI), a total scattering diffractometer at China Spallation Neutron Source (CSNS), Dongguan, China. The powder samples (petroleum coke-based carbon before and after discharge) were sealed in cylindrical ZrTi alloy containers with a diameter of 9 mm inside a glove box filled with helium gas. The sealed containers were then placed in a vacuum chamber equipped with an automated sample changing environment. The neutron wavelength range was 0.1 Å to 4.5 Å. The total scattering data was processed using the Mantid program to perform the data reduction. The data were normalized using vanadium tube and converted to the *S*(*Q*) function. The reduced pair distribution function *G*(*r*) was obtained by the Fourier transform of *S*(*Q*) which was merged in the *Q* range from 0.45 Å^-1^ to 43 Å^-1^.

*Electrochemical Measurement*

The working electrode was composed of 80 wt.% active materials, 10 wt.% Super P and 10 wt.% binders (LA133+CMC). The above materials were mixed in deionized water to form a homogeneous slurry, which was then coated on copper foil and dried at 100 °C for 12 h under vacuum. The average loading of the active material in the working electrode is around 1.5 mg cm^-2^. The CR2032 coin cells used in the electrochemical test were assembled in an argon-filled glove box using 1 M NaPF_6_ in ethylene carbonate (EC)/dimethyl carbonate (DMC) (1:1 v/v) as electrolyte, glass fiber (Whatman, GF/F) as separator and sodium foil as counter electrode. Cyclic voltammetry (CV) was conducted on CHI 660E electrochemical workstation at different scan rates within the voltage range of 0.01-3.00 V versus Na^+^/Na. The galvanostatic charge-discharge (GCD) tests were applied between 0.01 and 3.00 V at different current densities using a battery testing system (Neware CT4008). Galvanostatic intermittent titration technique (GITT) was performed at a current of 20 mA g^-1^ for 0.5 h with a relaxation time of 2 h.

*In situ XRD Analysis*

The in situ XRD analyses were conducted using the PANalytical X’Pert 3 with Cu *K*α radiation (*λ* = 0.15418 nm). The working electrode was composed of 80 wt.% active materials, 10 wt.% Super P and 10 wt.% binders. The mixed slurry was coated on the beryllium window and dried at 100 °C for 12 h under vacuum. The in situ XRD cell was assembled in an argon-filled glove box using the same separator, electrolyte and counter electrode as CR2032. The galvanostatic charge-discharge tests were conducted between 0.01 and 3.00 V at 25 mA g^-1^ using CHI 660E electrochemical workstation. Scanning in steps of 0.02 ° in the range of 10 °-45 °.

**Correlated Calculations**

*Scherer Equation*

$$\text{d}_{\text{002}}\text{=}\frac{\text{λ}}{\text{2sin}\text{θ}_{\text{002}}}$$

Where *λ* is the wavelength of the X-rays (0.15418 nm), 2*θ*_002_ is the reflection position of (002) reflection in the XRD pattern.

*Debye-Scherrer Equation*

$$\text{L}_{\text{a}}\text{=}\frac{\text{1.84}\text{λ}}{\text{B}_{\text{100}}\text{cos}\text{θ}_{\text{100}}}$$

$$\text{L}_{\text{c}}\text{=}\frac{\text{0.89}\text{λ}}{\text{B}_{\text{002}}\text{cos}\text{θ}_{\text{002}}}$$

Where *λ* is the wavelength of the X-rays (0.15418 nm), *B*_100_ and *B*_002_ are the full width at half maxima of the (100) and (002) reflections, 2*θ*_100_ and 2*θ*_002_ are the reflection position of the (100) and (002) reflections in the XRD pattern.

*True density*

As a reference point, graphite, regarded as a perfect layered materials without closed pores, possesses the highest true density value of 2.26 g cm^-3^. Therefore, the closed pore volume of carbon materials can be calculated using this equation:

$$\text{V}_{\text{closed pore}}\text{=}\frac{\text{1}}{\text{ρ}_{\text{true}}}\text{-}\frac{\text{1}}{\text{2.26}}$$

*Calculation of the Na^+^ Diffusion Coefficient for the GITT Tests (D_Na_^+^)*

The diffusion coefficients of Na^+^ (*D*_Na_^+^) can be calculated according to Fick 's second law equation as followed:

$$\text{D}_{\text{Na}^{\text{+}}}\text{=}\frac{\text{4}}{\text{πτ}}\left( \frac{\text{m}_{\text{B}}\text{V}_{\text{M}}}{\text{M}_{\text{B}}\text{S}} \right)^{\text{2}}\left( \frac{{\text{Δ}\text{E}}_{\text{S}}}{{\text{Δ}\text{E}}_{\text{τ}}} \right)^{\text{2}}$$

Where *τ* is the pulse duration, *m*_B_, *V*_M_ and *M*_B_ are mass load of electrode active materials, molar volume and molar mass, respectively. *S* is the active area of the electrode. Δ*E*_S_ and Δ*E*_τ_ are the potential difference of two adjacent steady-states and the voltage change due to the pulse current, respectively.

**Computational Methods**

All calculations based on density functional theory (DFT) were carried out within the projector augmented wave (PAW) method, as implemented in the using the DS-PAW^[1]^ module of Device Studio. The generalized gradient approximation (GGA)^[2]^ with the function of Perdew-Burke-Ernzerhof (PBE)^[3]^ was adopted to describe the exchange-correlation function. For all calculation, Van der Waals Correction based on DFT-D3.^[4,5]^ The convergence tolerance for the total energy and residual Hellmann-Feynman force were set to 10-5 eV per formula unit and 0.02 eV/Å, respectively. The cut-off energy was set to be 450 eV for the plane wave expansion. 1×1×1 *k*-point mesh was used to the integration in the irreducible Brillouin zone for structure optimization and 3×3×1 *k*-point mesh was used to the calculation of free energy. The basic simulation parameters of climbing image nudged elastic band (CI-NEB)^[6,7]^ calculations align with those used for structural optimization and four images were constrained while searching the intermediate.

After adjusting the interlayer spacing of the carbon material to different intervals, all carbon and hydrogen atoms are fixed. The model of carbon materials contains 192 carbon atoms and 16 hydrogen atoms. After adjusting the interlayer spacing of carbon materials to different interlayer distances, all carbon and hydrogen atoms are fixed. The number of sodium atoms is adjusted according to the calculation requirements.

**Supplementary Figures and Tables**


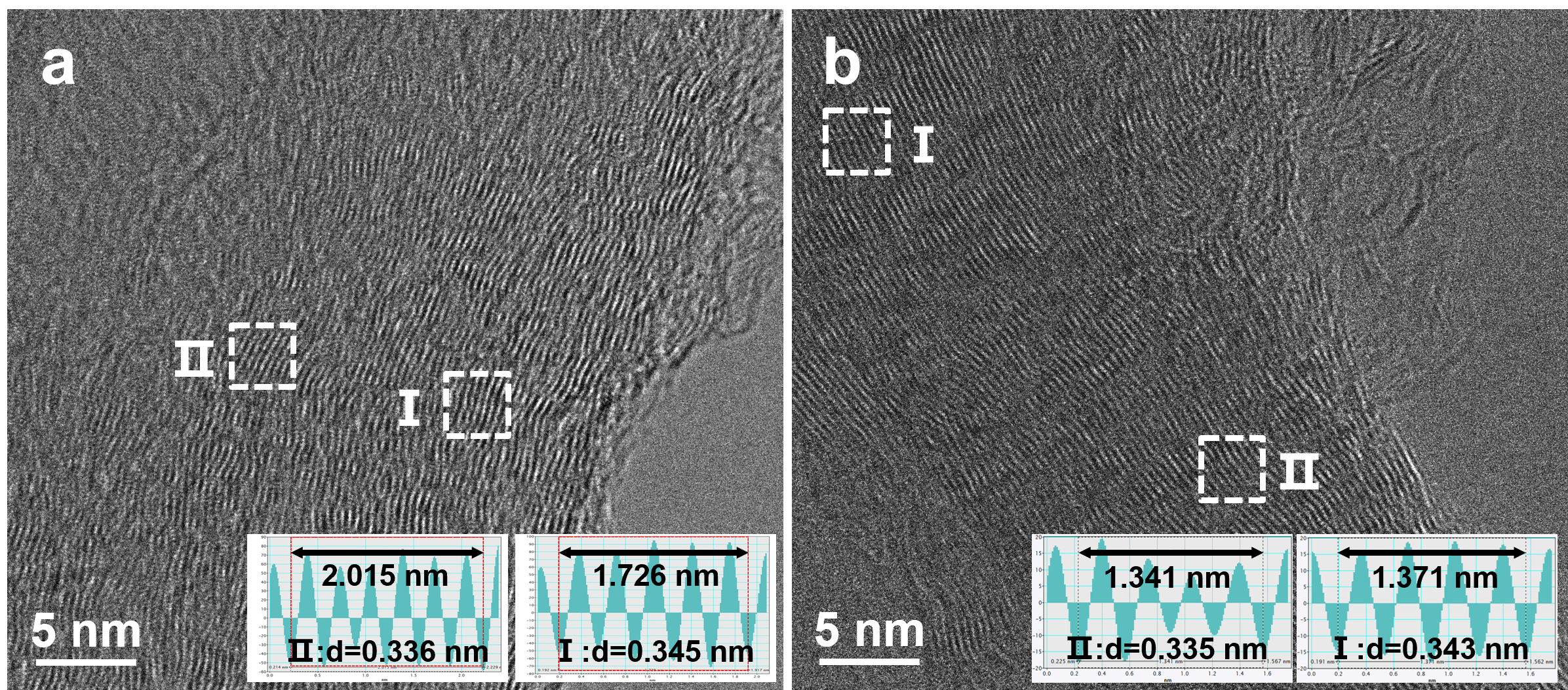


**Figure S1.** HRTEM images of a) PC-1400 and b) PC-1600.


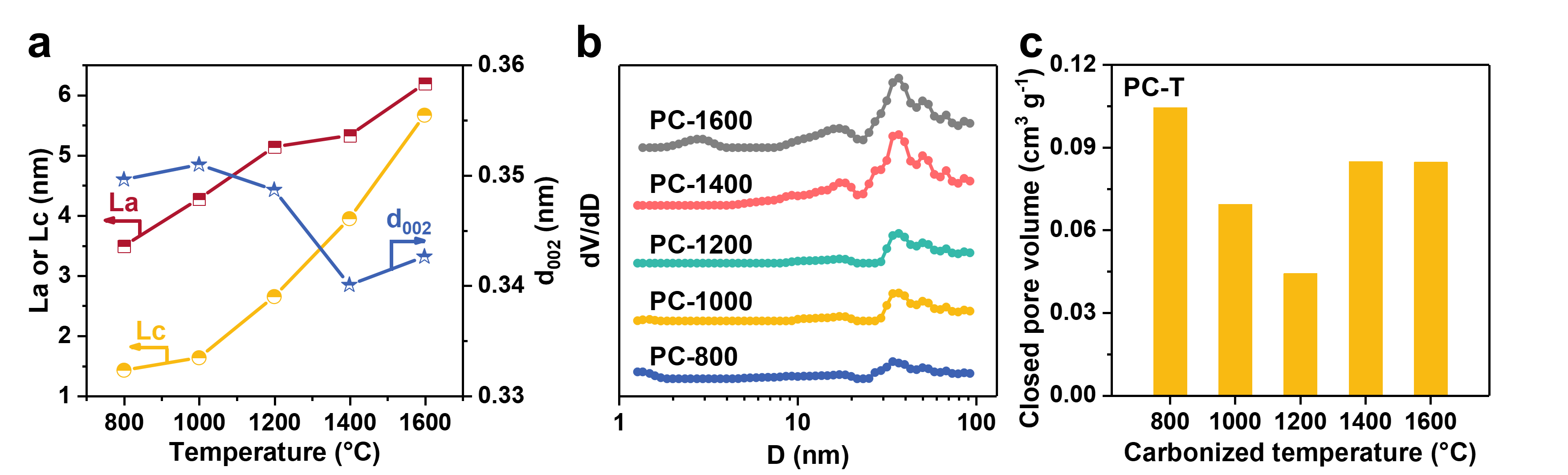


**Figure S2.** a) Relation curves of *d*_002_, *L*c, and *L*a and carbonization temperatures based on the XRD test, b) the corresponding pore size distributions, c) volume change trend of the closed pores.


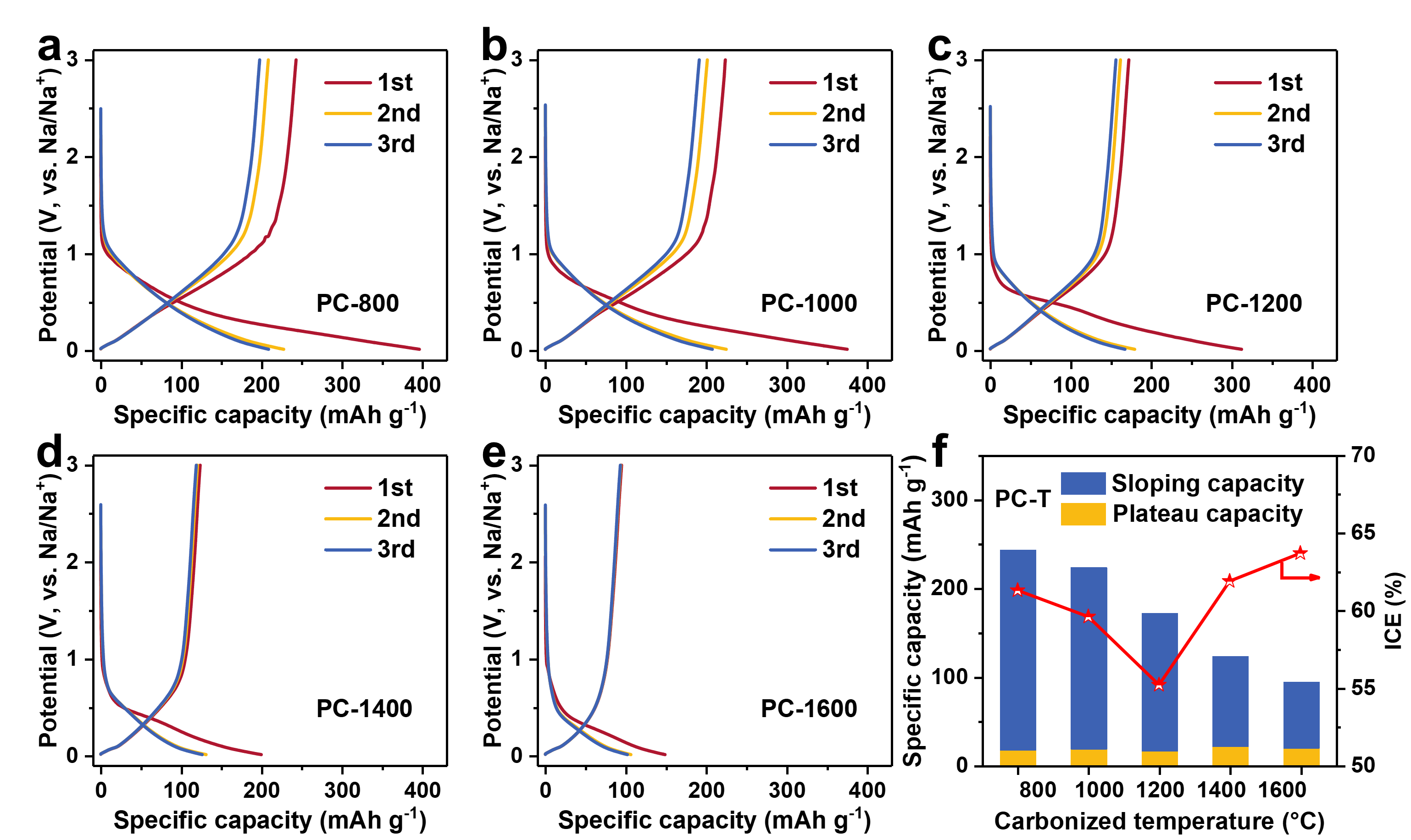


**Figure S3.** a-e) Charge/discharge profiles of the initial three cycles and f) the sloping capacity and plateau capacity of PC-T samples at 20 mA g^-1^.


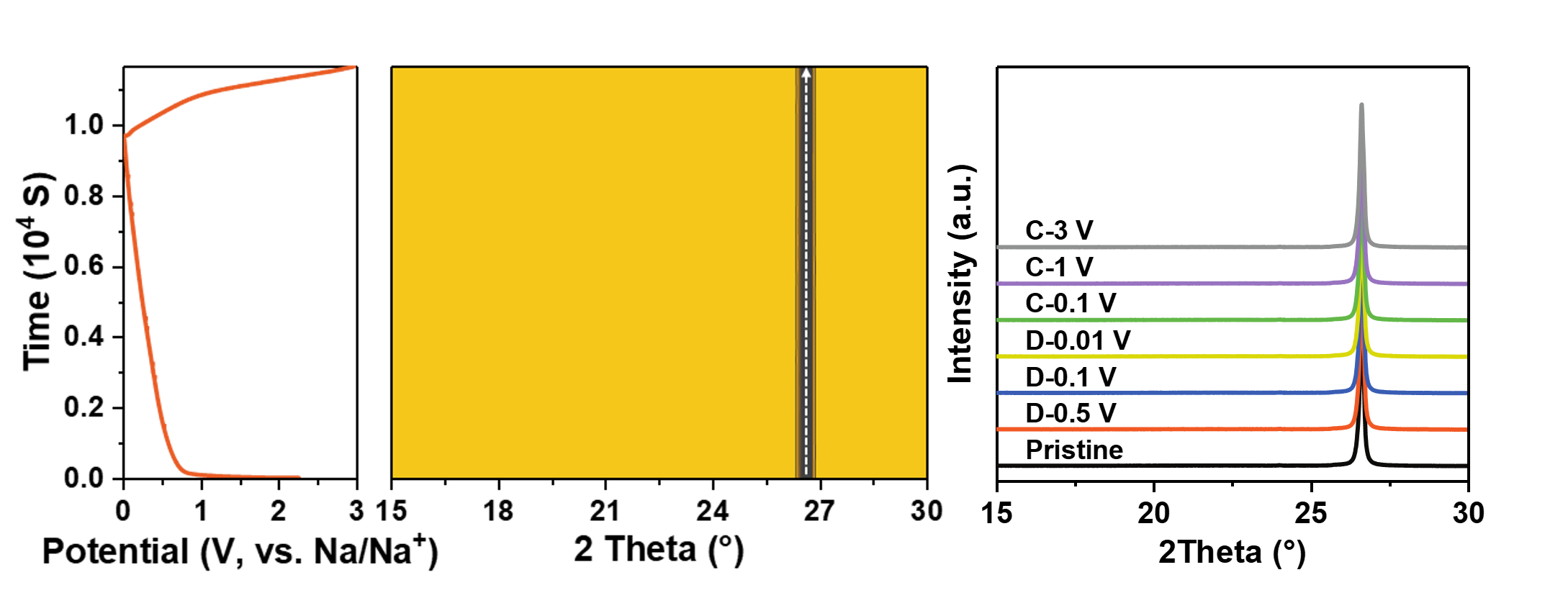


**Figure S4.** In situ XRD patterns during the first discharge-charge process for graphite.


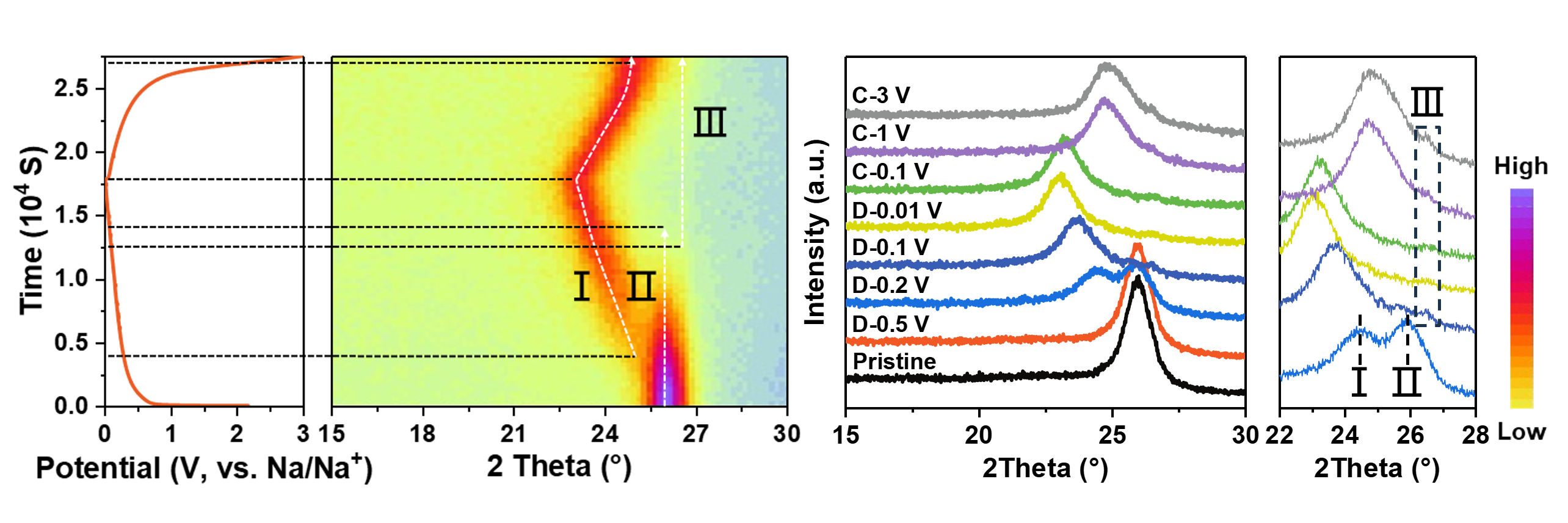


**Figure S5.** In situ XRD patterns during the first discharge-charge process for PA-1600. The figure on the right shows a local amplification of the XRD patterns.


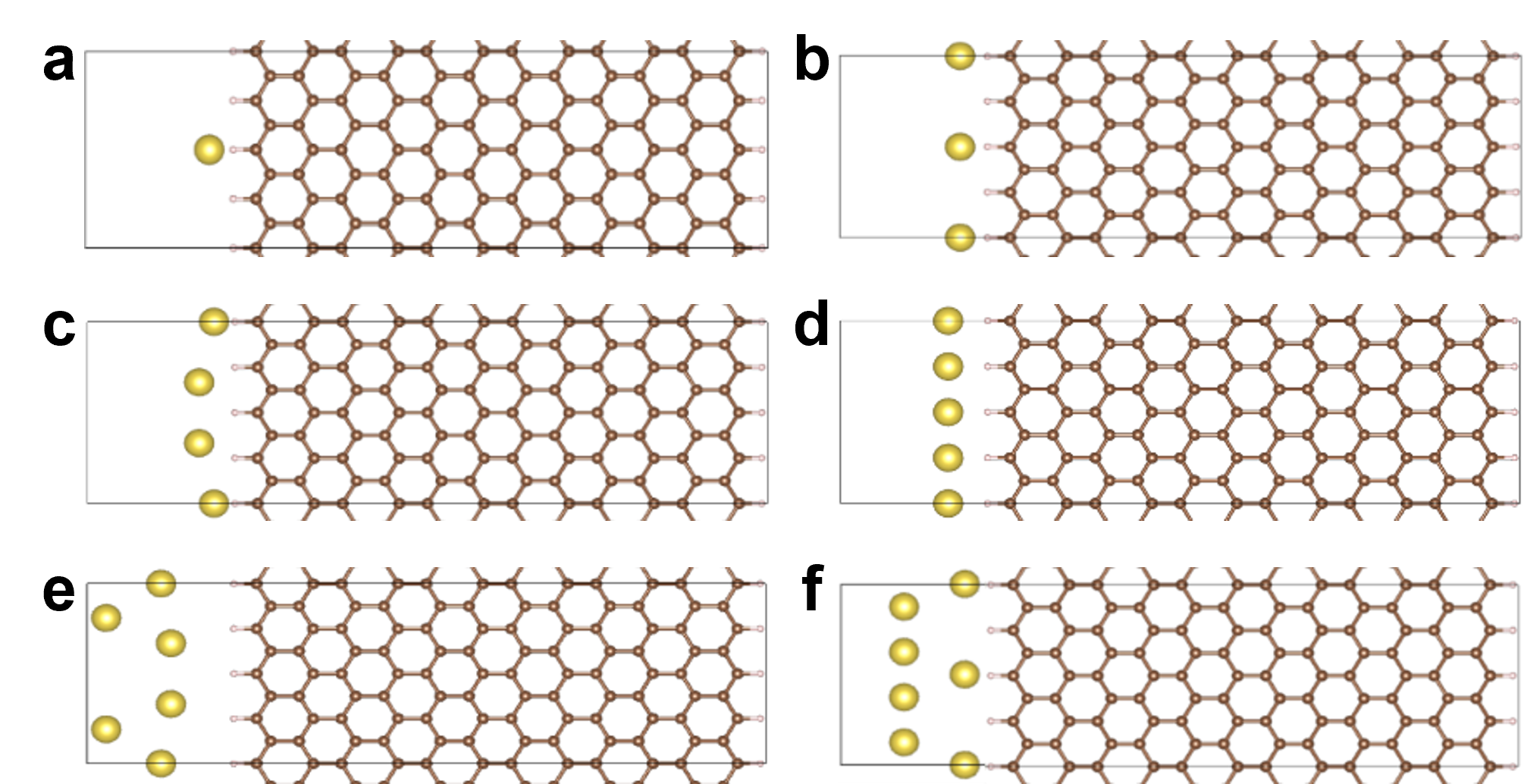


**Figure S6.** The optimized adsorption models at different edge coverages of sodium ions.

We constructed the adsorption models with 1-6 sodium ions for various interlayer spacing as the adsorption state, and the optimization results of structures are shown in Figure S6. We found that the adsorption models with more than 4 sodium ions are not stable. The excessive sodium ions will rearrange at the interface, and carbon materials cannot adsorb all sodium ions on the edges. Therefore, we defined the model of edge adsorption of 4 sodium ions as 100% coverage, while edge adsorption with 1, 2, and 3 sodium ions corresponded to 25%, 50%, and 75% coverage, respectively.


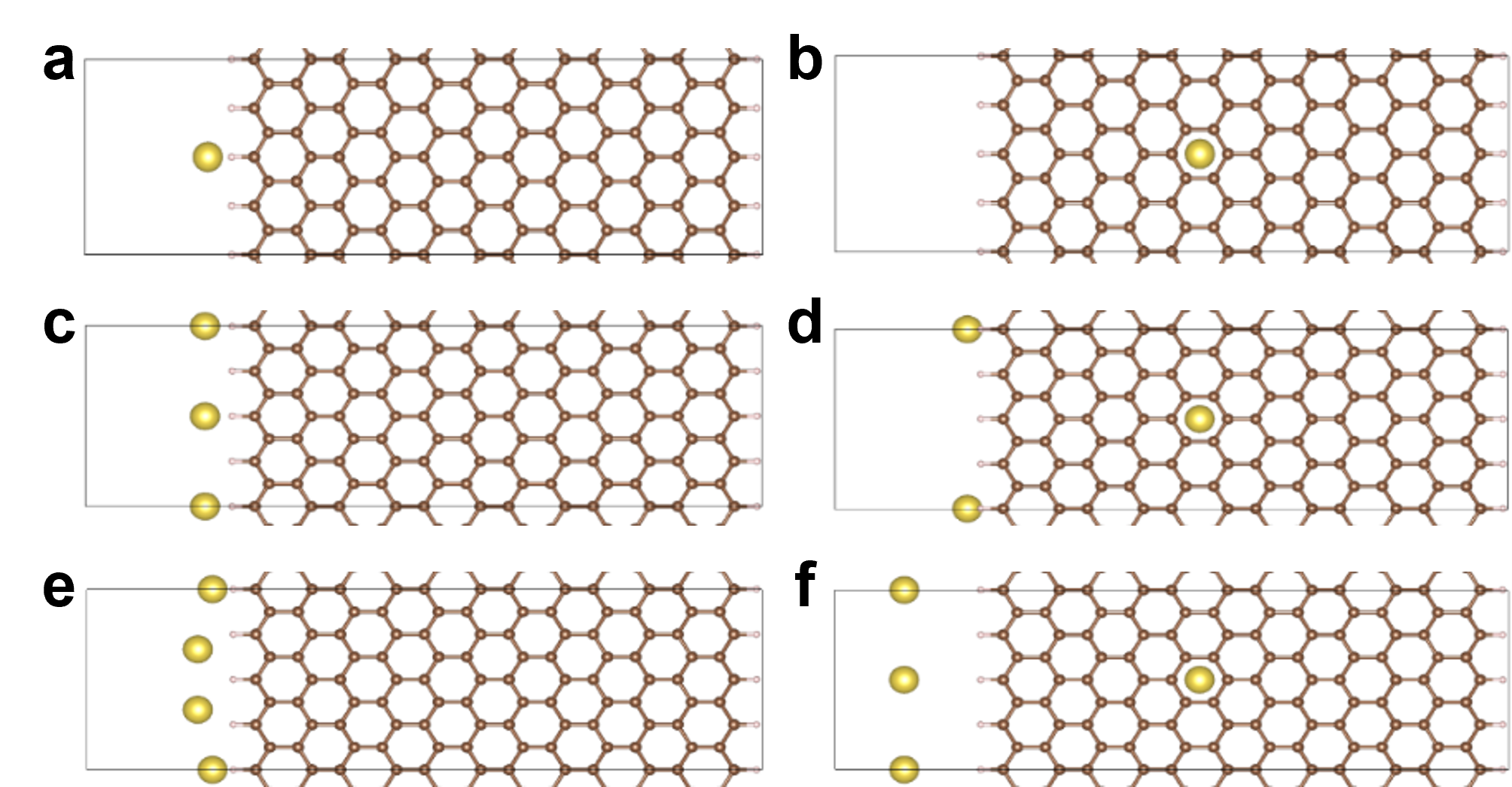


**Figure S7.** The calculation models of sodium adsorption and insertion state with edge coverages of a, b) 25%, c, d) 50% and e-f) 75%.


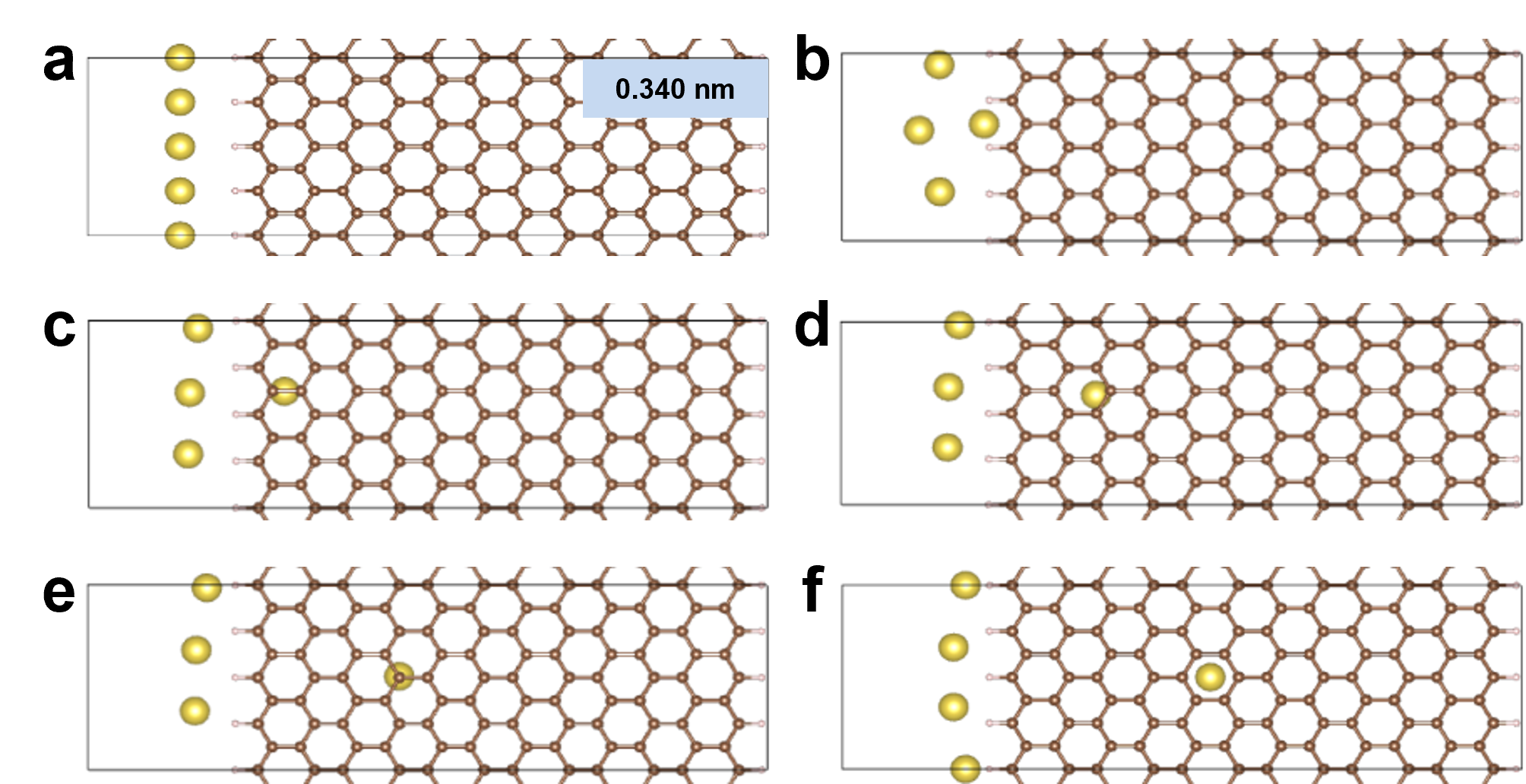


**Figure S8.** The sodium ions diffusion paths in the carbon model with interlayer spacing of 0.340 nm.


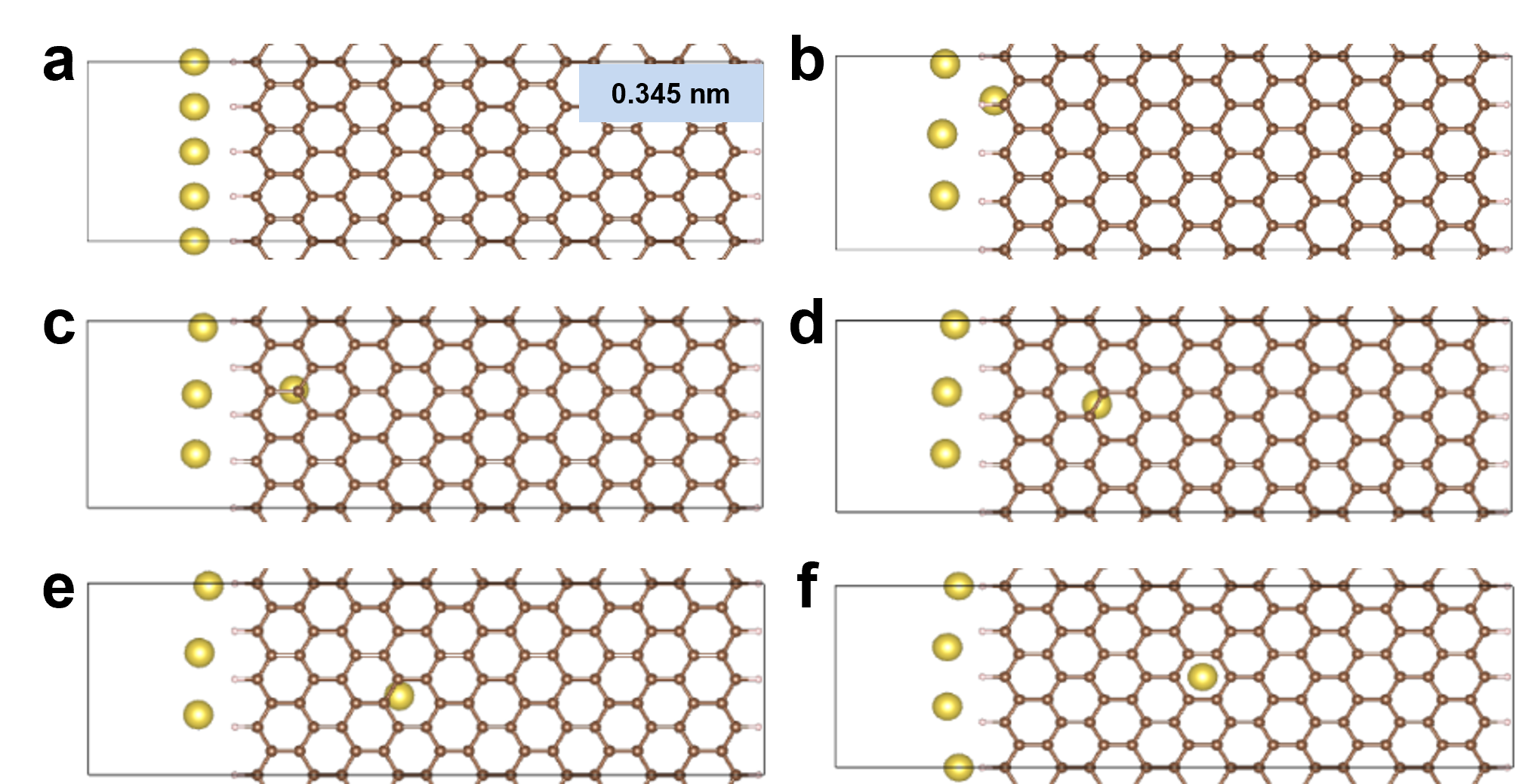


**Figure S9.** The sodium ions diffusion paths in the carbon model with interlayer spacing of 0.345 nm.


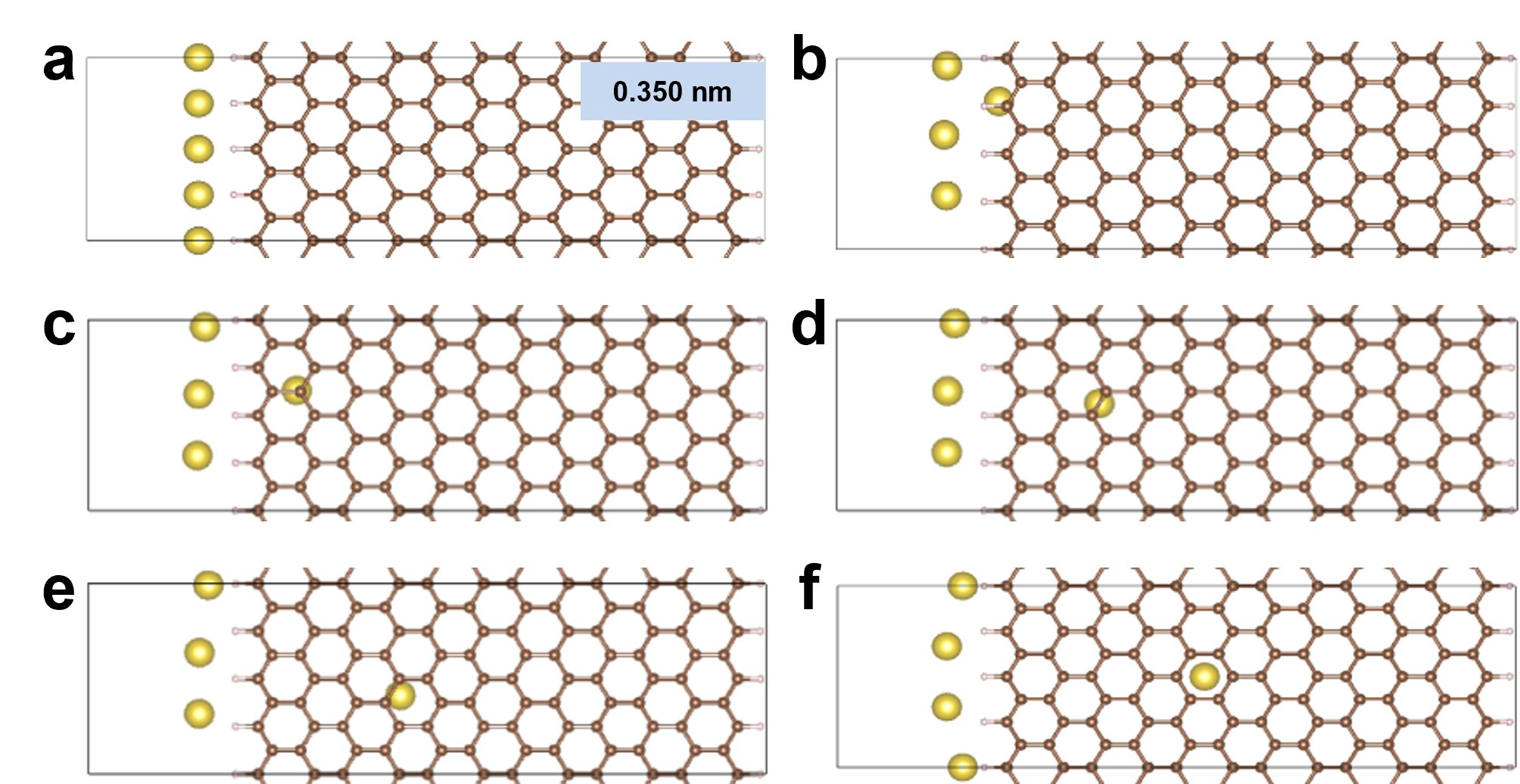


**Figure S10.** The sodium ions diffusion paths in the carbon model with interlayer spacing of 0.350 nm.


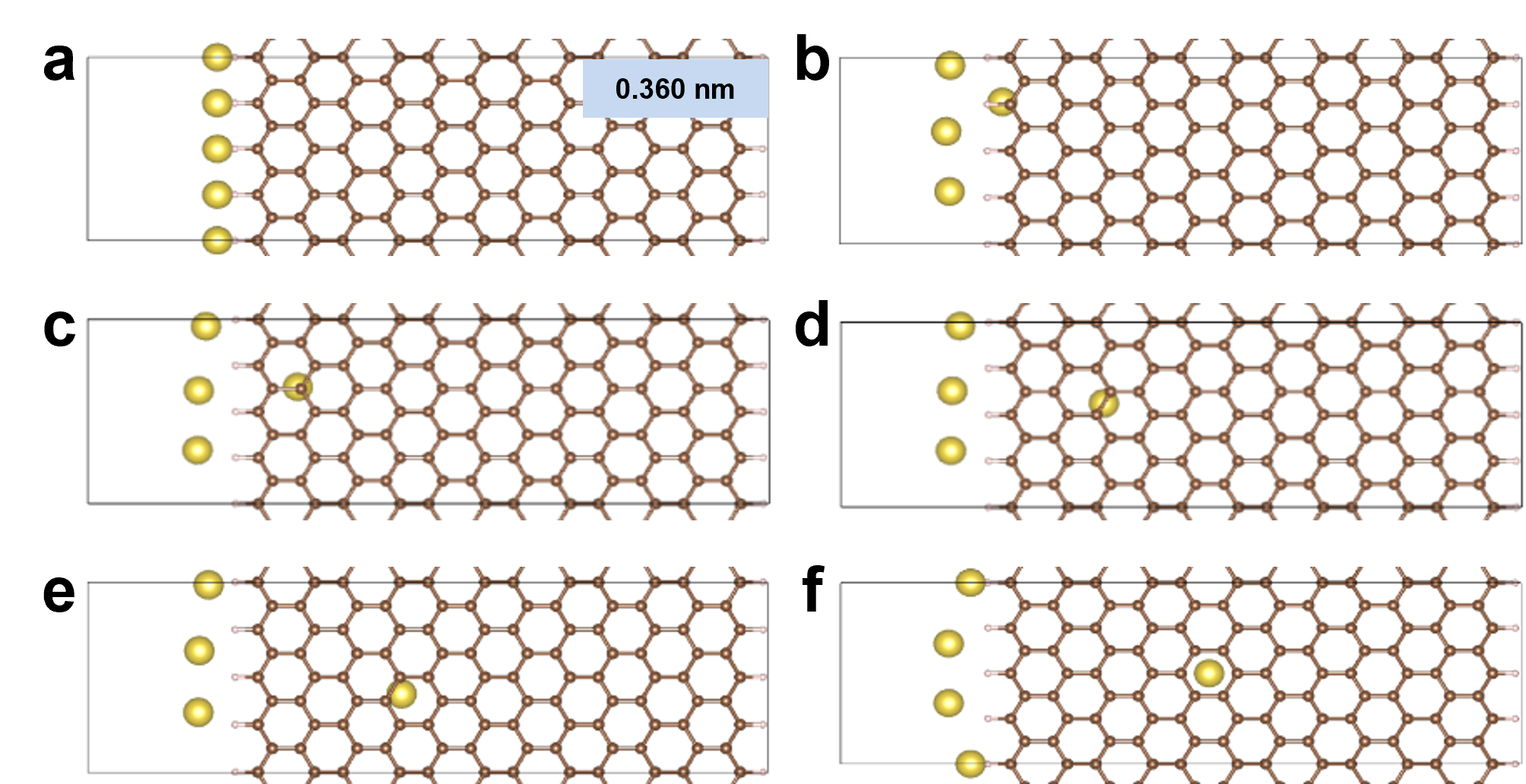


**Figure S11.** The sodium ions diffusion paths in the carbon model with interlayer spacing of 0.360 nm.


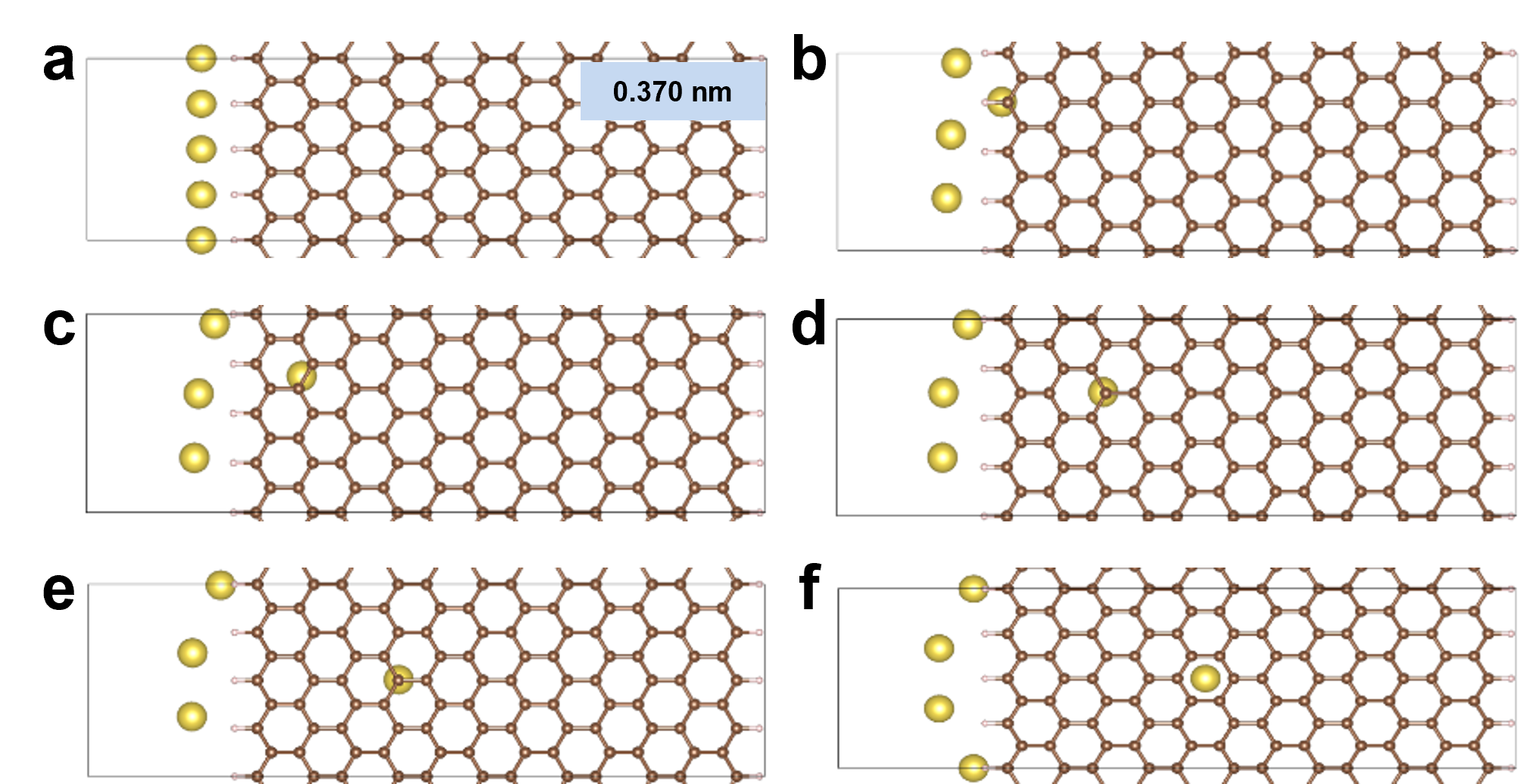


**Figure S12.** The sodium ions diffusion paths in the carbon model with interlayer spacing of 0.370 nm.


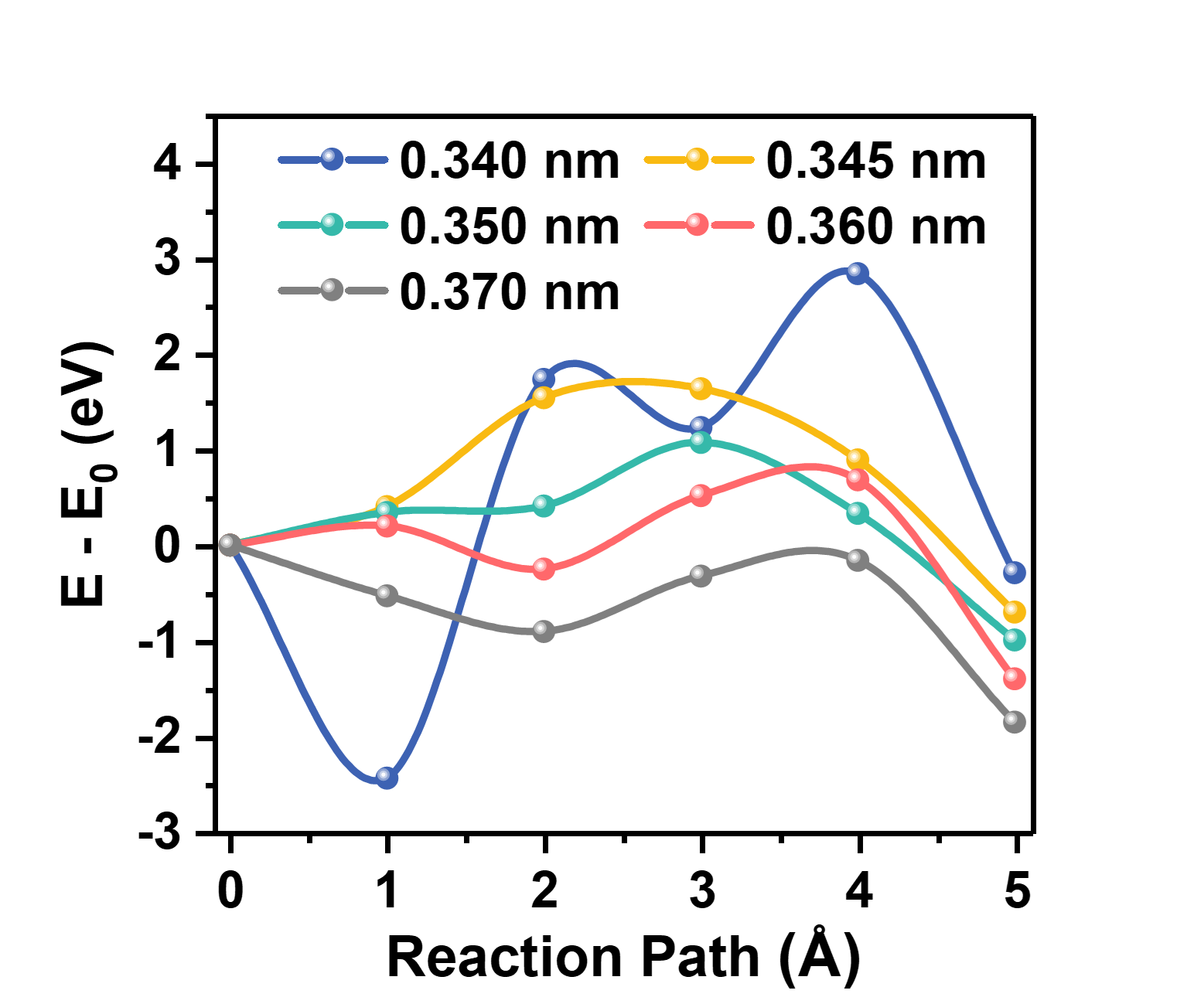


**Figure S13.** The intermediate state energies of sodium ions between different diffusion regions.


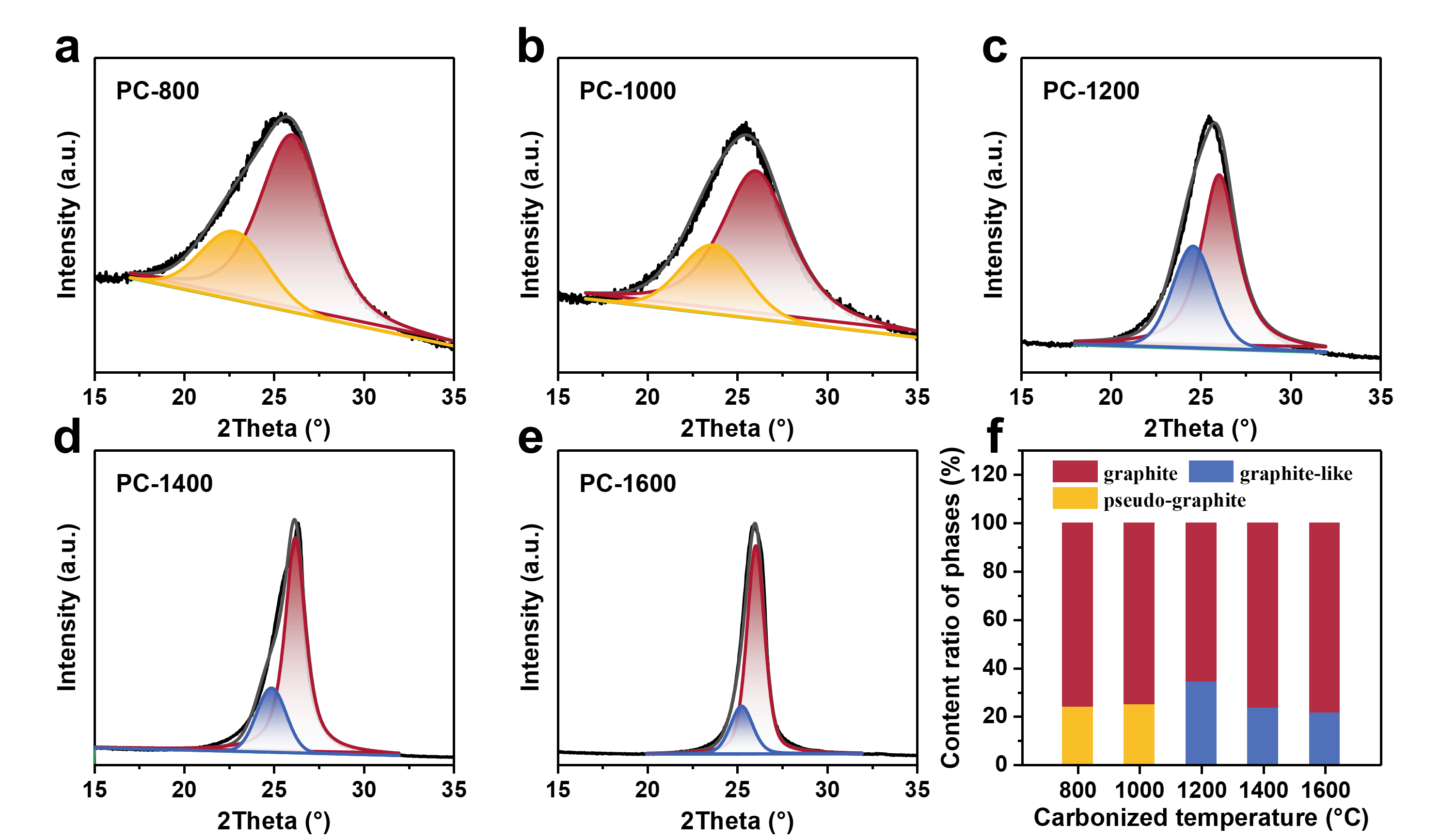


**Figure S14.** a-e) The (002) reflection-fitting for PC-T and f) corresponding content ratio of each microcrystalline phase.


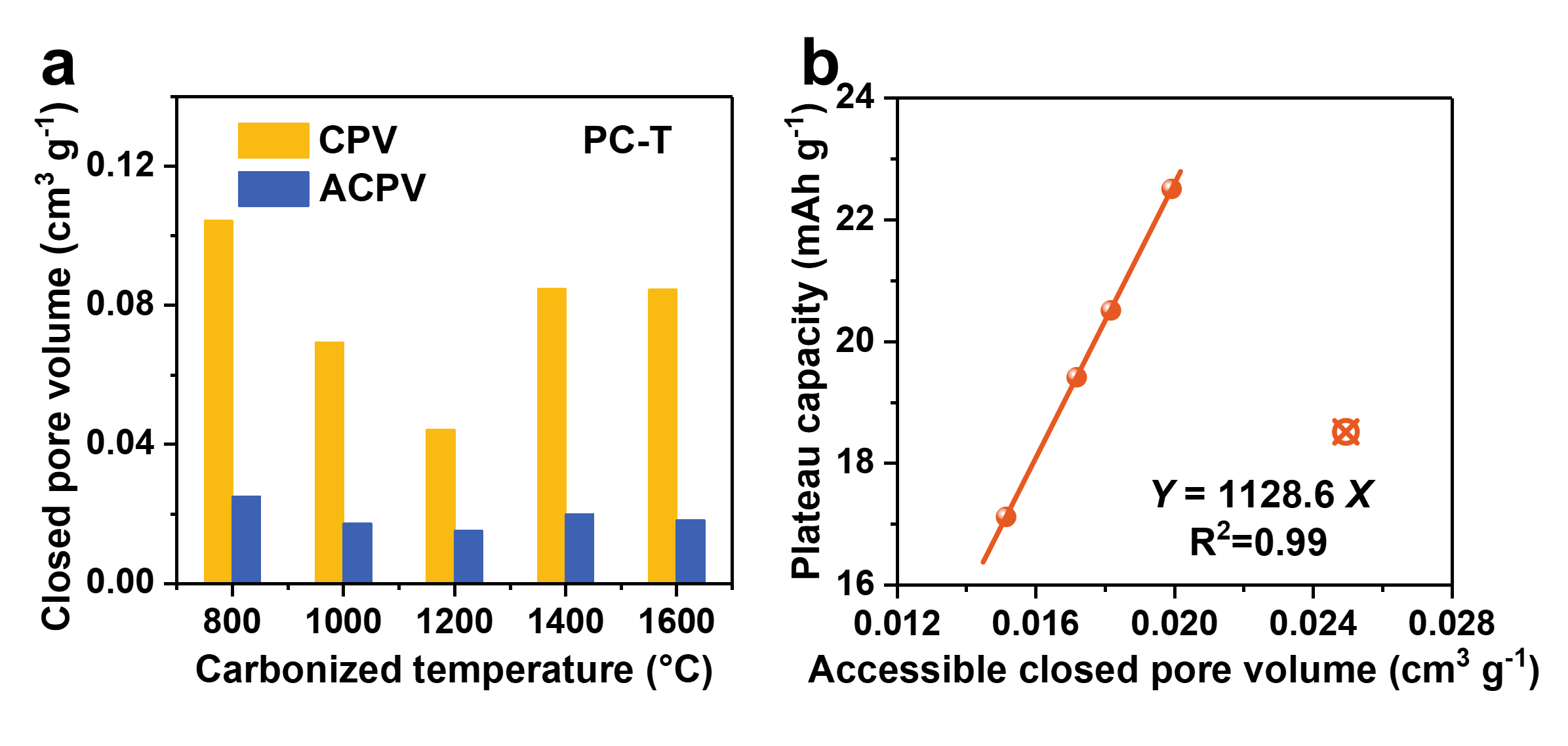


**Figure S15.** a) Closed pore volume (CPV) and accessible closed pore volume (ACPV) of PC-T. b) Correlation curves between the plateau capacity and the accessible closed pore volume.


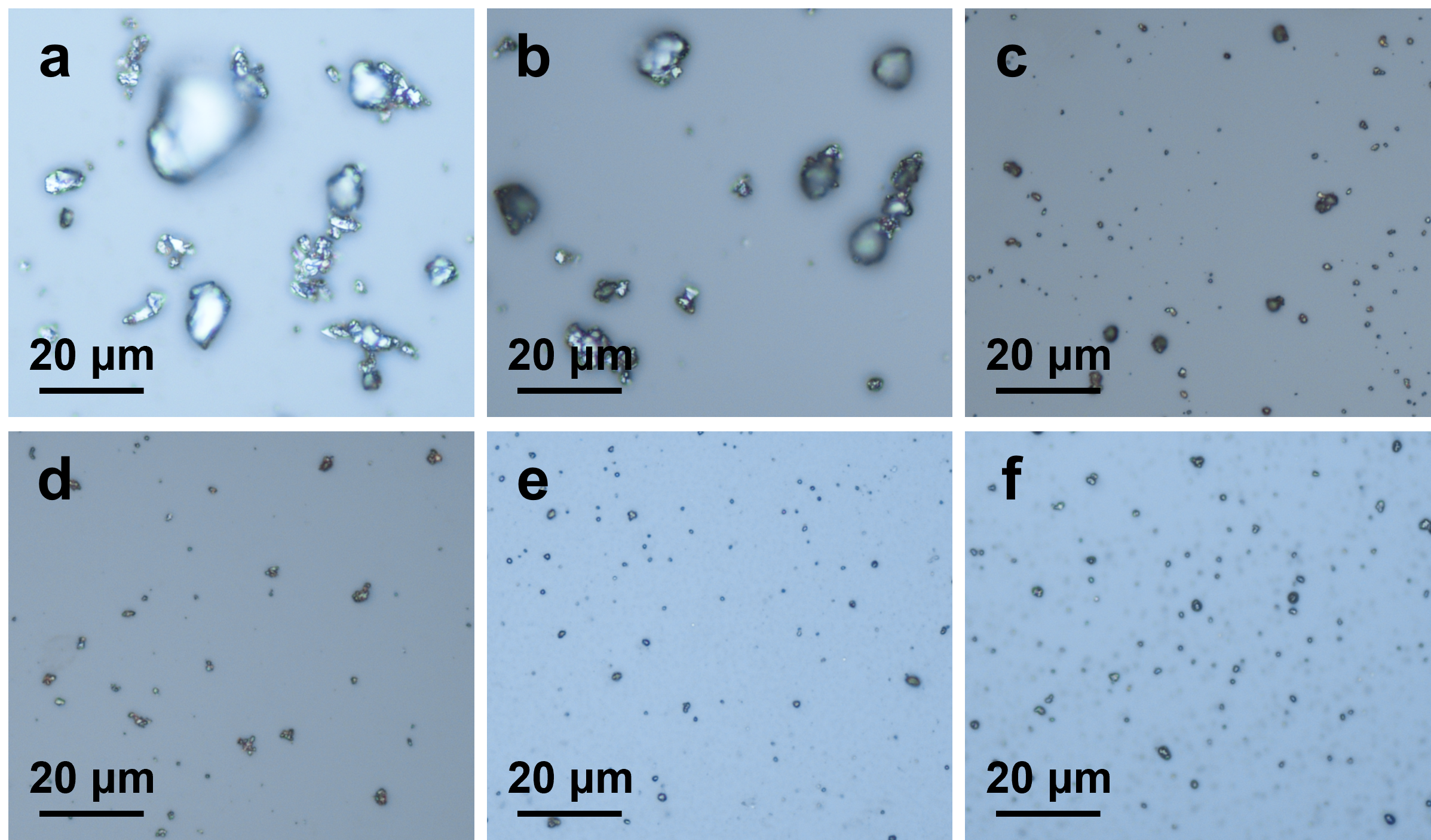


**Figure S16.** Optical microscopy images of a) PC, b) PC-0.1 h, c) PC-1 h, d) PC-2 h, e) PC-3 h, f) PC-4 h.


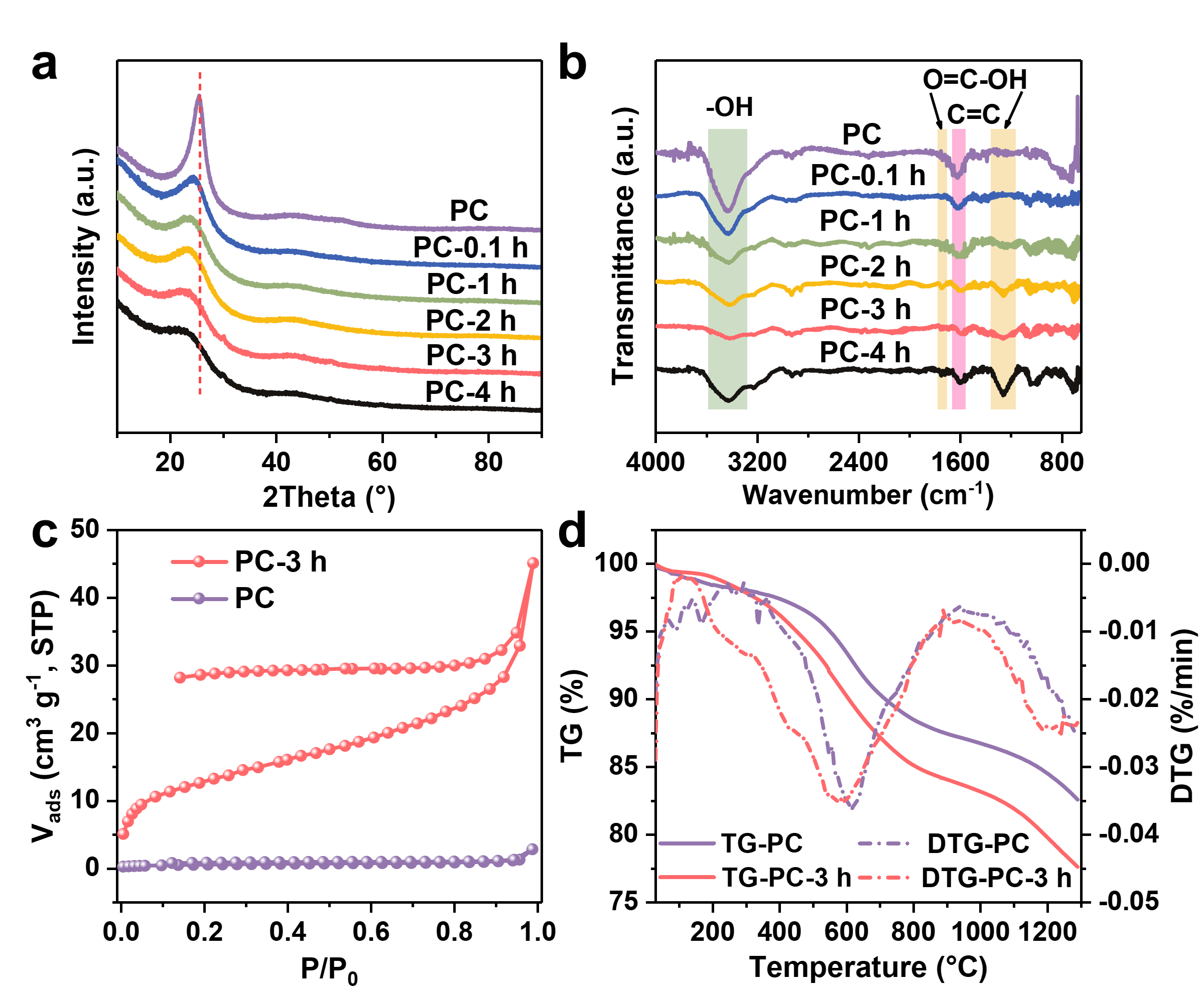


**Figure S17.** a) XRD patterns and b) FTIR spectra of petroleum coke treated with different ball-milling time, c) N_2_ adsorption-desorption isotherms and d) TG-DTG curves of PC and PC-3 h.


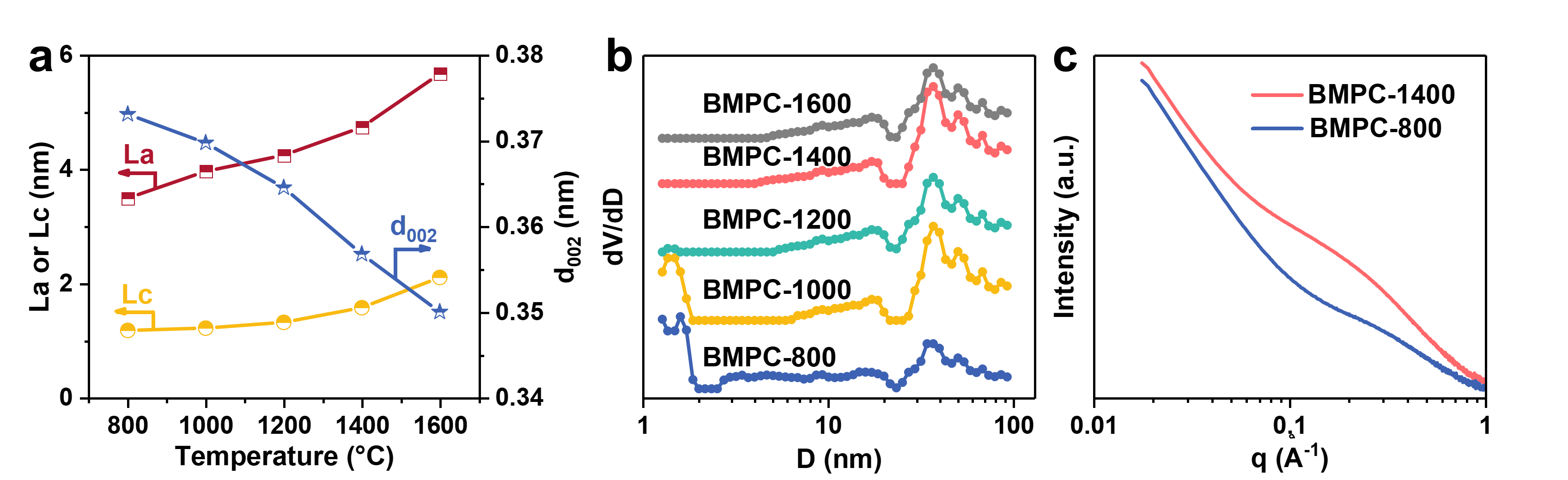


**Figure S18.** a) Relation curves of *d*_002_, *L*c, and *L*a and carbonization temperatures based on the XRD test, b) the corresponding pore size distributions for BMPC-T, c) SAXS patterns of BMPC-800 and BMPC-1400.


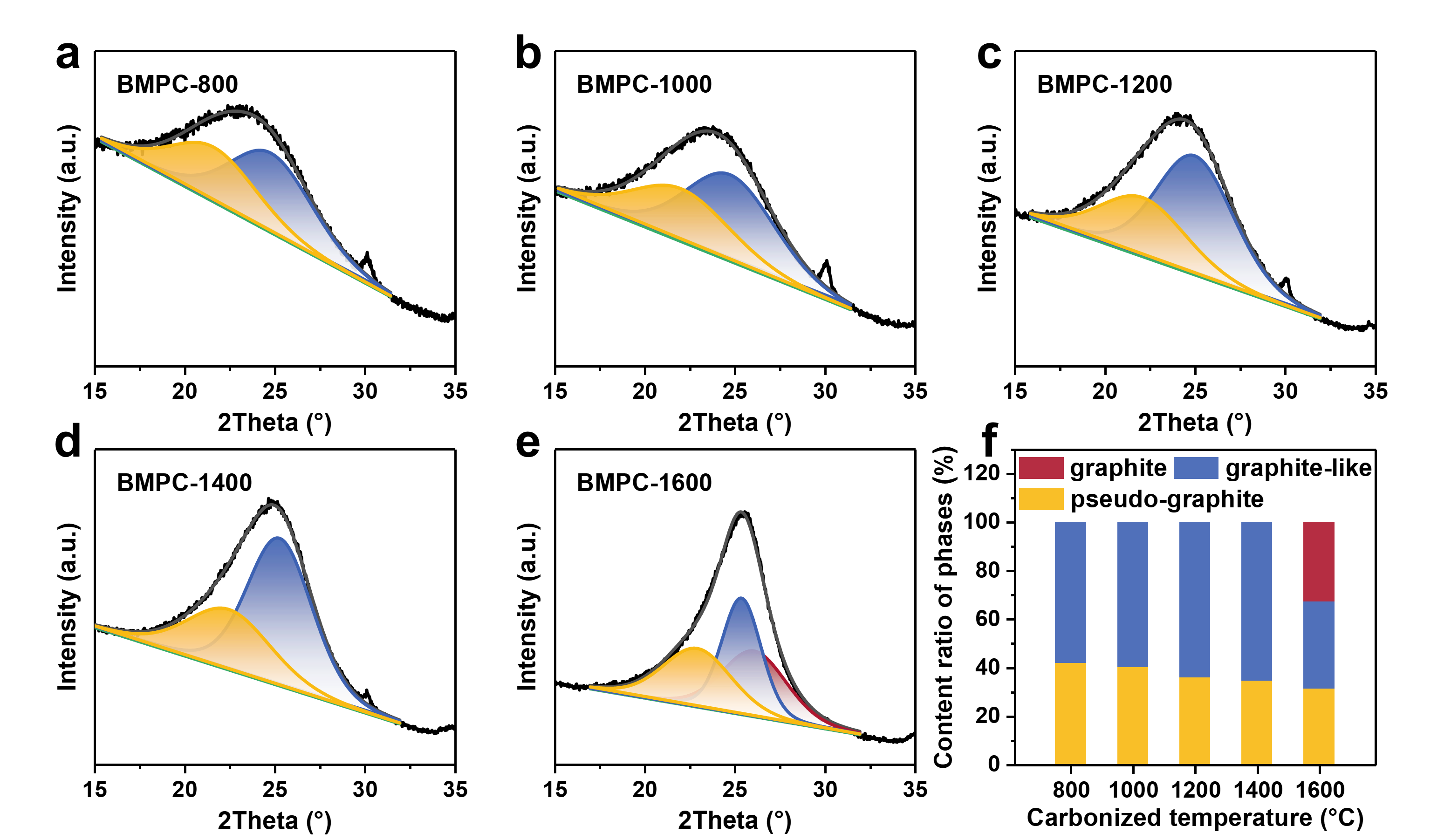


**Figure S19.** a-e) The (002) reflection-fitting for BMPC-T and f) the corresponding content ratio of each microcrystalline phase.


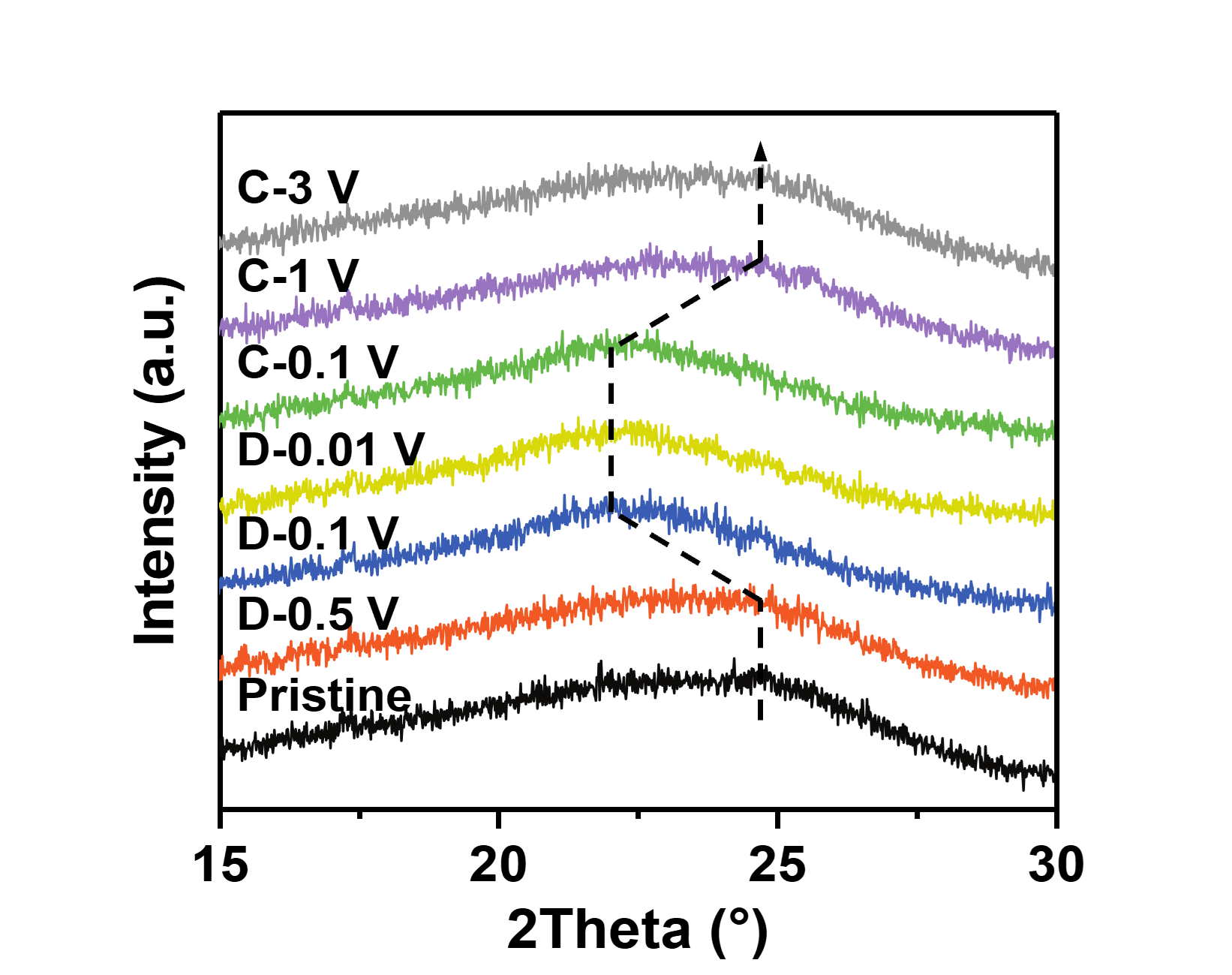


**Figure S20.** In situ XRD patterns during the first discharge-charge process for BMPC-1400.


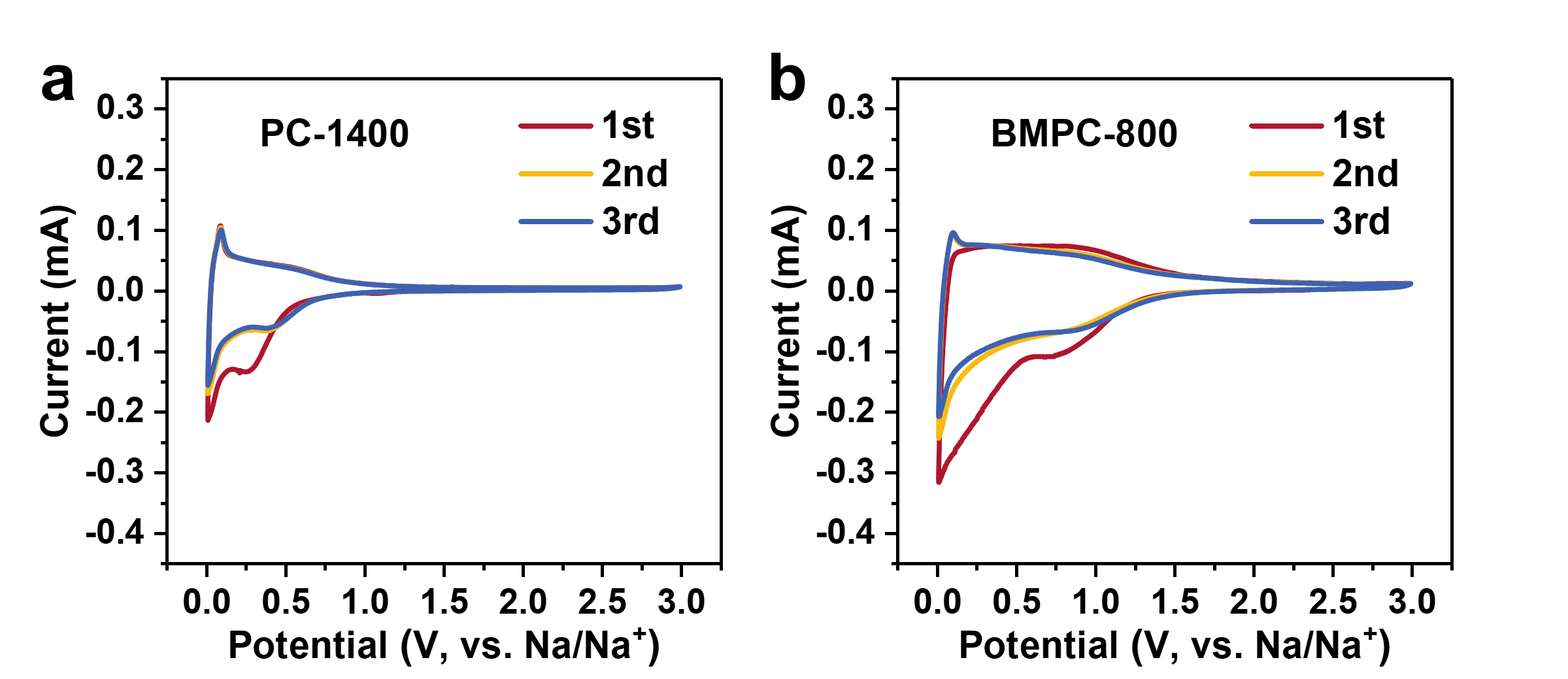


**Figure S21.** CV curves of a) PC-1400 and b) BMPC-800 at the voltage window of 3.0-0.01 V recorded at a scan rate of 0.1 mV s^-1^.


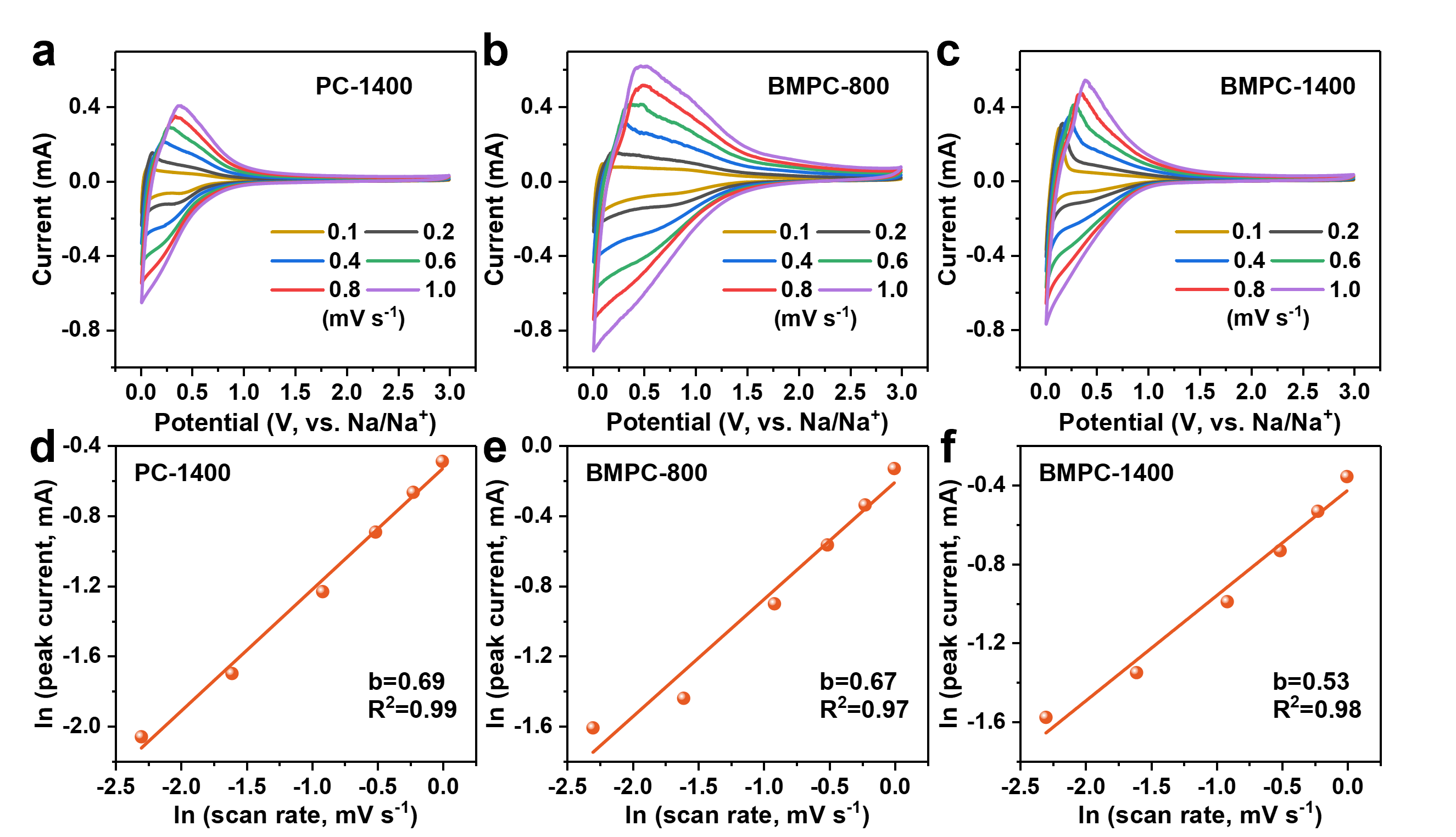


**Figure S22.** CV curves of a) PC-1400, b) BMPC-800 and c) BMPC-1400 at various scan rates. The linear correlation between the current peak and the scan rate of d) PC-1400, e) BMPC-800 and f) BMPC-1400.


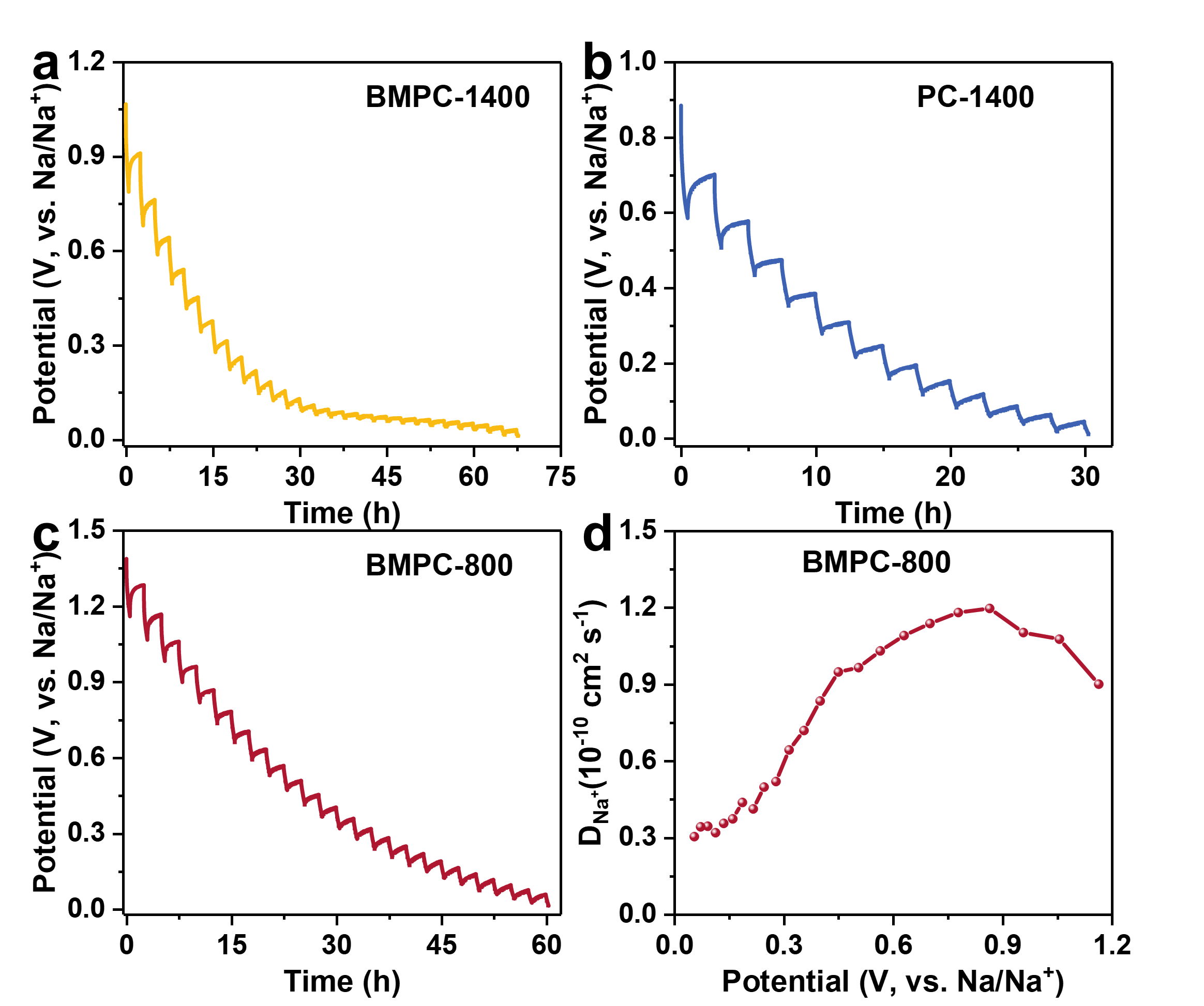


**Figure S23.** The galvanostatic intermittent titration technique (GITT) profiles of a) BMPC-1400, b) PC-1400 and c) BMPC-800 during the discharge process, d) the variation of calculated sodium ions diffusion coefficients against different potentials for BMPC-800.


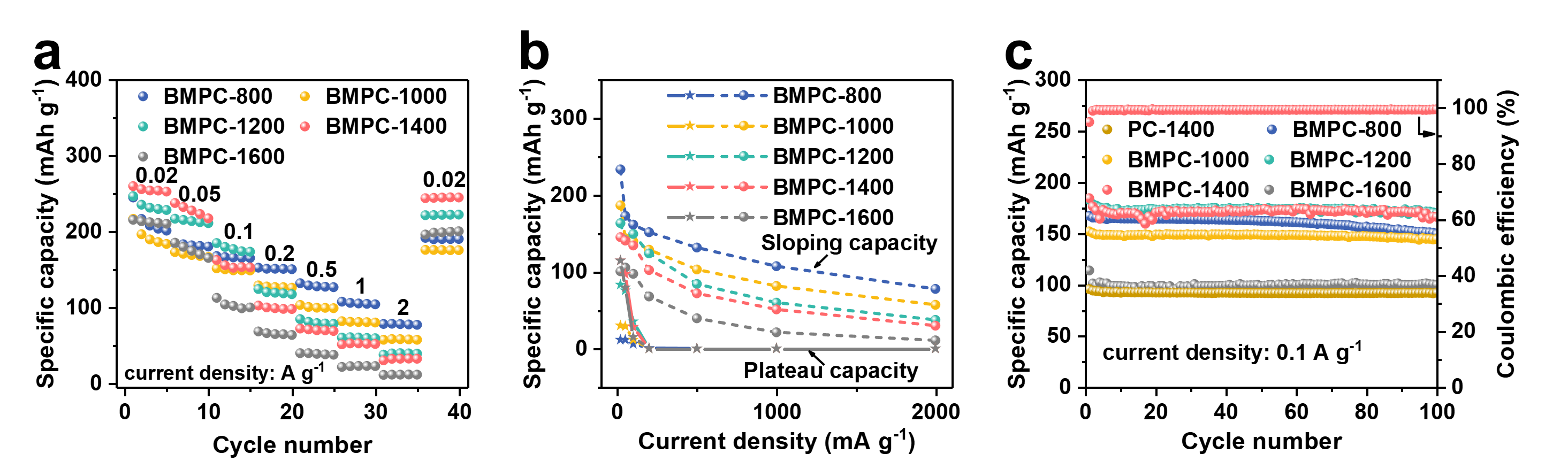


**Figure S24.** a) The rate performance, b) the capacity retention of sloping and plateau region under different current densities and c) cycling performance at 100 mA g^-1^ of BMPC-T.


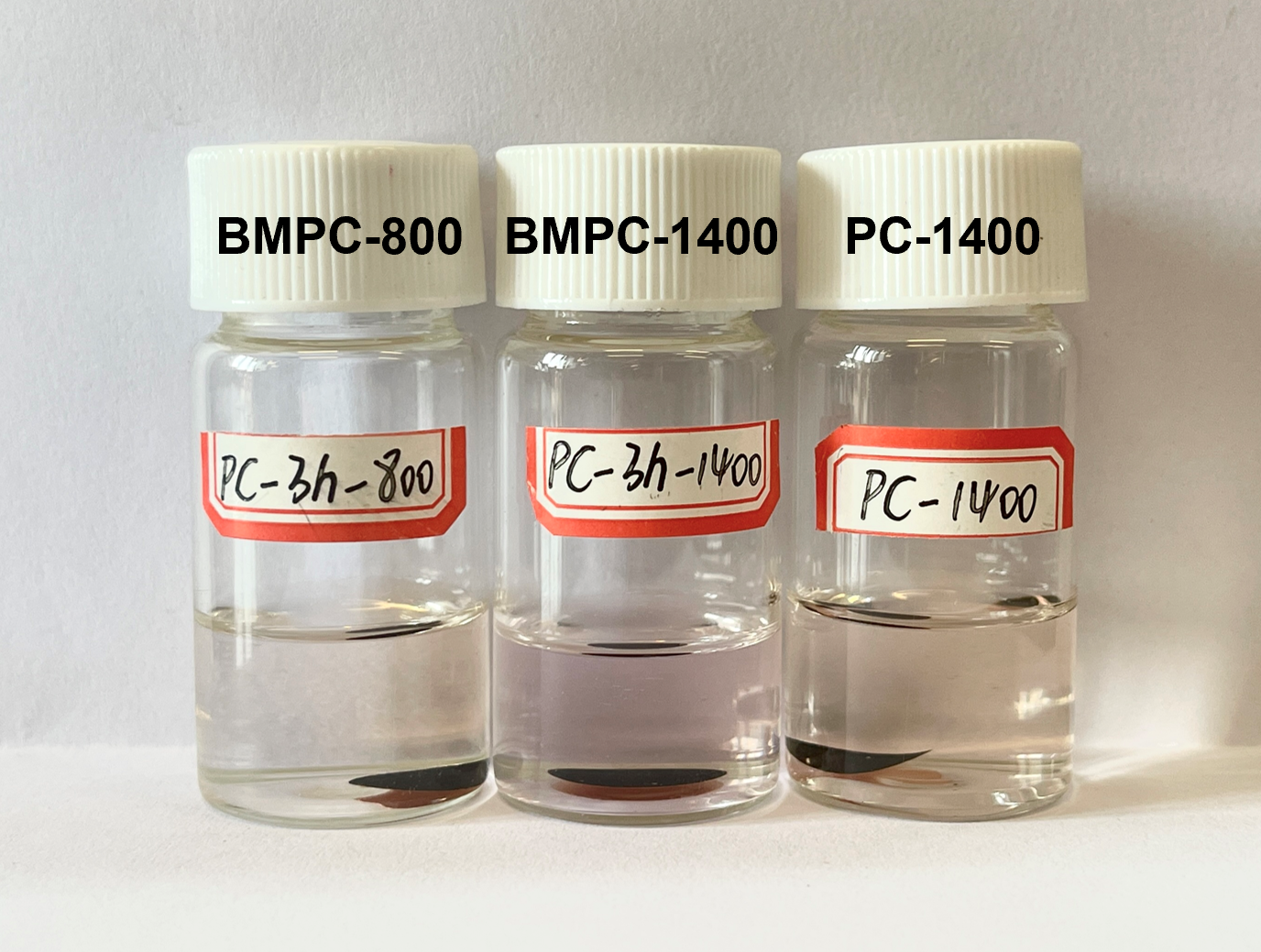


**Figure S25.** The reaction phenomena between electrodes after discharge to 0.01 V and the ethanol containing 1% phenolphthalein.

**Table S1.** The elemental analysis and proximate analysis of the petroleum coke.

| Sample | Elemental analysis | | | | | Proximate analysis | | | |
| --- | --- | --- | --- | --- | --- | --- | --- | --- | --- |
|  | C_daf_/% | H_daf_/% | S_daf_/% | N_daf_/% | O_daf_/% | M_ar_/% | A_ar_/% | V_ar_/% | FC_ar_/% |
| PC | 87.2 | 3.3 | 7.6 | 1.4 | 0.5 | 3.8 | 0.5 | 10.0 | 85.7 |

**Table S2.** The structural parameters and pore structure information of PC-T and BMPC-T.

| Sample | *d*_002_  (nm) | *L*_c_  (nm) | *L*_a_  (nm) | *S*_BET_  (m^2^ g^-1^) | *V*_open pore_  (cm^3^ g^-1^) | True density  (g cm^-3^) | *V*_closed pores_  (cm^3^ g^-1^) |
| --- | --- | --- | --- | --- | --- | --- | --- |
| PC-800 | 0.350 | 1.42 | 3.49 | 4.1 | 0.003 | 1.83 | 0.104 |
| PC-1000 | 0.351 | 1.63 | 4.27 | 3.1 | 0.003 | 1.95 | 0.069 |
| PC-1200 | 0.349 | 2.65 | 5.14 | 1.2 | 0.002 | 2.05 | 0.044 |
| PC-1400 | 0.340 | 3.94 | 5.33 | 4.2 | 0.023 | 1.90 | 0.085 |
| PC-1600 | 0.343 | 5.66 | 6.19 | 4.8 | 0.020 | 1.90 | 0.085 |
| BMPC-800 | 0.373 | 1.18 | 3.50 | 103.4 | 0.063 | 2.20 | 0.012 |
| BMPC-1000 | 0.370 | 1.22 | 3.97 | 141.8 | 0.072 | 2.11 | 0.031 |
| BMPC-1200 | 0.365 | 1.32 | 4.25 | 16.4 | 0.024 | 2.00 | 0.058 |
| BMPC-1400 | 0.357 | 1.57 | 4.74 | 12.4 | 0.023 | 1.83 | 0.105 |
| BMPC-1600 | 0.350 | 2.11 | 5.67 | 10.3 | 0.020 | 1.67 | 0.158 |

**Table S3.** The XRD (002) reflection fitting analysis for PC-T and BMPC-T samples.

|  | graphite phase | | |  | graphite-like phase | | |  | pseudo-graphite phase | | | ACPC  content/% |
| --- | --- | --- | --- | --- | --- | --- | --- | --- | --- | --- | --- | --- |
|  | 2*θ*/° | *d*_002_/nm | Area/% |  | 2*θ*/° | *d*_002_/nm | Area/% |  | 2*θ*/° | *d*_002_/nm | Area/% |  |
| PC-800 | 26.05 | 0.342 | 76.1 |  | - | - | - |  | 22.82 | 0.390 | 23.9 | 23.9 |
| PC-1000 | 26.05 | 0.342 | 75.2 |  | - | - | - |  | 23.69 | 0.375 | 24.8 | 24.8 |
| PC-1200 | 26.05 | 0.342 | 65.7 |  | 24.60 | 0.362 | 34.3 |  | - | - | - | 34.3 |
| PC-1400 | 26.21 | 0.340 | 76.5 |  | 24.88 | 0.358 | 23.5 |  | - | - | - | 23.5 |
| PC-1600 | 26.05 | 0.342 | 78.5 |  | 25.26 | 0.352 | 21.5 |  | - | - | - | 21.5 |
| BMPC-800 | - | - | - |  | 24.82 | 0.359 | 58.1 |  | 21.60 | 0.411 | 41.9 | 100 |
| BMPC-1000 | - | - | - |  | 24.75 | 0.360 | 60.0 |  | 22.03 | 0.403 | 40.0 | 100 |
| BMPC-1200 | - | - | - |  | 25.01 | 0.356 | 64.2 |  | 22.11 | 0.402 | 35.8 | 100 |
| BMPC-1400 | - | - | - |  | 25.29 | 0.352 | 65.5 |  | 22.47 | 0.396 | 34.5 | 100 |
| BMPC-1600 | 26.10 | 0.341 | 32.9 |  | 25.39 | 0.351 | 35.9 |  | 22.93 | 0.388 | 31.2 | 67.1 |

Reference

[1] P. E. Blochl, *Phys Rev B Condens Matter* **1994**, *50*, 17953.

[2] G. Kresse, J. Furthmuller, *Phys Rev B Condens Matter* **1996**, *54*, 11169.

[3] J. P. Perdew, K. Burke, M. Ernzerhof, *Phys. Rev. Lett.* **1996**, *77*, 3865.

[4] S. Grimme, J. Antony, S. Ehrlich, H. Krieg, *J. Chem. Phys.* **2010**, *132*, 154104.

[5] S. Grimme, S. Ehrlich, L. Goerigk, *J. Comput. Chem.* **2011**, *32*, 1456.

[6] G. Henkelman, G. Jóhannesson, H. Jónsson, *Theor. Chem. Phys.* **2002**, *5*, 269.

[7] R. A. Olsen, G. J. Kroes, G. Henkelman, A. Arnaldsson, H. Jónsson, *J. Chem. Phys.* **2004**, *121*, 9776.
